# Molecular insights into Atorvastatin’s role in delaying intervertebral disc degeneration

**DOI:** 10.3389/fcell.2025.1693951

**Published:** 2025-12-11

**Authors:** Yupeng Li, Linghao Wang, Xingjie Wang, Kang Li, Qian Wang, Kai Gao, Cunxin Zhang, Chaoliang Lv

**Affiliations:** 1 Department of Spine Surgery, Jining NO.1 People’s Hospital Affiliated to Shandong First Medical University and Shandong Academy of Medical Sciences, Jining, China; 2 Jining Medical University, Jining, China; 3 Shandong First Medical University and Shandong Academy of Medical Sciences, Jinan, China; 4 Shandong Provincial Key Medical and Health Laboratory of Neuroinjury and Repair, Jining, China; 5 Department of Orthopaedics, Jining No. 1 People’s Hospital Affiliated to Shandong First Medical University and Shandong Academy of Medical Sciences, Jining, China; 6 Medical Integration and Practice Center, Shandong University, Jining, China

**Keywords:** atorvastatin, Nrf-2, oxidative stress, disc degeneration, nucleus pulposus cells

## Abstract

**Introduction:**

This study aimed to investigate the molecular mechanisms and effects of atorvastatin (Ator) in delaying intervertebral disc degeneration (IDD), with a particular focus on its role in modulating oxidative stress and apoptosis in nucleus pulposus cells (NPCs) via the Nrf2 signaling pathway.

**Methods:**

*In vitro*, rat NPCs were treated with different concentrations of atorvastatin, and the optimal concentration was determined using the Cell Counting Kit-8 (CCK-8) assay. Reactive oxygen species (ROS) levels were measured using a ROS detection kit, while apoptosis was evaluated by flow cytometry and TUNEL assays. Western blotting was performed to assess the expression of nuclear factor erythroid 2–related factor 2 (Nrf2), its downstream antioxidant proteins, and apoptosis-related proteins. *In vivo*, fifteen rats were randomly assigned to control, acupuncture, and atorvastatin injection groups. After four weeks of treatment, intervertebral discs were collected for histological evaluation using hematoxylin-eosin (HE) and Flip-Red O staining. Apoptosis within the discs was further examined by electron microscopy.

**Results:**

Pretreatment with 10 μM atorvastatin significantly improved the survival rate of NPCs exposed to hydrogen peroxide (H_2_O_2_) and reduced apoptosis. Atorvastatin enhanced the antioxidant capacity of NPCs and decreased intracellular ROS accumulation. It promoted Nrf2 activation and upregulated the expression of downstream antioxidant factors, thereby exerting a protective effect against oxidative stress–induced injury. Inhibition of Nrf2 attenuated these protective effects of atorvastatin. Moreover, atorvastatin reduced the expression of apoptosis-related proteins and inhibited H_2_O_2_-induced extracellular matrix degradation in chondrocytes. In the *in vivo* rat model, atorvastatin treatment ameliorated histological signs of intervertebral disc degeneration and reduced apoptosis in disc tissues.

**Discussion:**

These findings indicate that atorvastatin alleviates H_2_O_2_-induced oxidative stress and apoptosis in NPCs predominantly through activation of the Nrf2 signaling pathway, leading to preservation of extracellular matrix integrity and attenuation of disc degeneration. Collectively, the data support the potential of atorvastatin as a therapeutic agent for the prevention or treatment of lumbar intervertebral disc degeneration.

## 1 Introduction

With the aging of the population in our society, intervertebral disc degeneration (IDD) has become a significant cause of lower back pain and other intervertebral disc diseases (Mertimo et al., 2022), which not only seriously affects the quality of life of patients but also brings excellent economic pressure (Vergroesen et al., 2015). However, due to the complexity of its pathogenesis, there are minimal methods to delay or reverse disc degeneration. Current studies have found that abnormalities in nucleus pulposus cells (NPC) are a critical factor in the development of IDD. The phenotype of NPC changes with age (Li et al., 2022). The decrease in aggregated proteoglycans and type Ⅱ collagen, as well as the increase in metalloproteinase expression, leads to a dysfunctional balance between synthesis and catabolism, destruction of the extracellular matrix and apoptosis of the nucleus pulposus, which ultimately triggers IDD (Zhang et al., 2021). Therefore, protecting NPC is essential for maintaining disc stability (Sun et al., 2021). Therefore, the security of NPC is necessary to maintain disc stability.

A complete antioxidant system exists inside the cell, in which nuclear factor-erythroid 2-related factor 2 (Nrf2) and its downstream antioxidant proteins play a crucial role in maintaining cellular oxidative homeostasis. Under normal conditions, Nrf2 and Keap1 bind in the cytoplasm, are inactive, and are ubiquitinated and degraded by the proteasome. When oxidative stress occurs, Nrf2 detaches from Keap1. It rapidly moves toward the nucleus (Weiss-Sadan et al., 2023; Ghareghomi et al., 2023), which subsequently acts on antioxidant response elements (ARE) in the nucleus and then activates transcriptional pathways and their downstream expression of antioxidant proteins (Zhang et al., 2025) such as heme oxygenase-1(HO-1), NAD(P) H quinone dehydrogenase 1 (NQO-1), superoxide dismutase (SOD), to alleviate cytotoxicity caused by oxidative stress and restore cellular redox homeostasis (Wang et al., 2025; Xiang et al., 2022). In addition, it has also been shown that Nrf2 enhances cellular detoxification and assists cells in clearing harmful substances (Verza et al., 2025). Nrf2 has also been shown to improve cellular detoxification and assist cells in clearing toxic substances.

Atorvastatin (Ator) is one of the most common competitive inhibitors of 3-hydroxy 3-methylglutaryl coenzyme A reductase, mainly used to treat hyperlipidemia-induced cardiovascular and cerebrovascular diseases (Ning et al., 2023). In recent years, it has been found that Ator has antioxidant, anti-inflammatory, and anti-apoptotic effects in addition to lowering blood lipids (Yu et al., 2022; Robinson et al., 2014; Werida et al., 2021). In recent years, studies have found that Ator reduces blood lipids and has antioxidant, anti-inflammatory, and anti-apoptotic effects. The antioxidant mechanism of Ator has received particular attention and has been widely studied. Its antioxidant mechanism mainly includes the following aspects: 1. Directly scavenging excessive intracellular reactive oxygen species (Kowalski et al., 2006); 2. Improve the activity of antioxidant enzymes, thus enhancing the scavenging ability of reactive oxygen species; 3. Enhance the level of antioxidants in the body, such as glutathione. It has been shown that statins can effectively inhibit the onset of disc degeneration. However, the mechanism remains unclear and controversial (Chen et al., 2023).

Although the potential role of statins in the treatment of intervertebral disc degeneration has been initially demonstrated, the differences in efficacy among various statins warrant further investigation. Compared to other statins, Ator exhibits unique advantages in both clinical and experimental studies. Firstly, Ator possesses stronger antioxidant properties, which are reflected not only in its direct scavenging ability against reactive oxygen species, but also in its enhancement of endogenous antioxidant enzyme systems (Deng et al., 2025). As a lipophilic statin, Ator can enter cells via passive diffusion and is widely distributed across various tissues, whereas hydrophilic statins require protein transporters to enter cells and exert their effects (Sokalska et al., 2018). Secondly, Ator surpasses other statins in terms of tissue permeability and bioavailability, enabling it to more effectively cross the blood–brain barrier and other tissue barriers, thereby exerting superior therapeutic effects in relatively ischemic tissues such as the intervertebral disc (Williams et al., 2023). Moreover, clinical studies have shown that Ator demonstrates outstanding anti-inflammatory effects and can more effectively inhibit the release of proinflammatory cytokines, which is particularly significant for diseases like intervertebral disc degeneration, where inflammation is a major pathological feature (Chen et al., 2025). Animal studies have also confirmed the chondroprotective effects of statins; intra-articular injection of statins in animal models of knee osteoarthritis can significantly suppress degeneration of cartilage tissue, maintain the structural integrity of chondrocytes, and reduce the expression of matrix metalloproteinases (Lee et al., 2025). Compared with other statins, Ator demonstrates stronger effects in protecting chondrocytes and promoting extracellular matrix synthesis, which may be related to its unique lipophilicity and superior tissue penetration (Hosseinzadeh et al., 2019). Based on these distinctive pharmacological properties and clinical benefits, we have selected Ator as the target drug for this study to explore its specific molecular mechanisms in the treatment of intervertebral disc degeneration.

In this study, we evaluated the ability of Ator to ameliorate H_2_O_2_ induced oxidative stress and extracellular matrix degradation in intervertebral discs and initially explored its mechanism of action. The results showed that Ator enhanced the expression of Nrf2 and its downstream-related antioxidant proteins, effectively inhibiting apoptosis and extracellular matrix degradation. Our findings imply that Ator may be a potential drug for treating intervertebral degenerative disc disease (IDD).

## 2 Materials and methods

### 2.1 Extraction and culture of rat NPC

NP tissue blocks were isolated from tissues of 12-week-old Sprague-Dawley (SD) rats, digested with 0.25% trypsin (gibco, US) for 25 min, terminated by digestion, then digested with 0.2% type Ⅱ collagenase (Solarbio, CN) for 25 min, filtered through a 0.75 μ m cell filter, and resuspended in culture medium containing 10% fetal bovine Cells were filtered through 0.75 μm cell filters, resuspended in medium containing 10% fetal bovine serum (gibco, US) and 1% antibiotics (gibco, US), and then incubated at 37 °C in an incubator containing 5% CO_2_. Passage cultures were performed when the cell concentration reached 80%–90% confluence. The first three generations of NPC were used for all experiments.

NPC were divided into five groups: control group (routine culture without any treatment), H_2_O_2_ group (induced using 300 um/mL H_2_O_2_), Ator treatment group (10 umol/mL Ator (MCE, US) and 300 umol/mL H_2_O_2_ co-treatment were selected), Nrf2 inhibitor group (10 μM Nrf2-IN-1 (MCE, US) plus 300 umol/mL H_2_O_2_ co-treatment), Nrf2 inhibitor plus Ator group (10uM Nrf2-IN-1 plus 300 umol/mL H_2_O_2_ and 10 umol/mL Ator co-treatment) Cells were incubated at 37 °C in a 5% CO_2_ incubator for subsequent experiments.

### 2.2 Cell viability assay

CCK-8 kit (Dojindo, Japan) was used to detect drug toxicity and cell viability. Different groupings of NPC were inoculated into 96-well cell culture plates at a density of 1000 cells per well in a final volume of 100 μL. The cells were treated with the drug after wall attachment, and then 10 μL CCK-8 reagent was added to each well. 2 h later, the absorbance values were measured at 450 nm using an enzyme marker (Bio-Tek, United States). Each experiment was performed in triplicate.

### 2.3 Reactive oxygen measurement

After drug treatment, cells were collected and suspended in diluted DCFH-DA (Beyotime, China) and incubated in a 37 °C cell culture incubator for 20 min. The mixing was inverted every 3–5 min to make complete contact between the probe and the cells. The cells were washed three times with a serum-free cell culture medium to remove the DCFH-DA that did not enter the cells fully. The cells were collected for on-line assay.

### 2.4 Western blotting

Cellular proteins were extracted (performed on ice) using the Total Protein Extraction Kit (Beyotime, China) and the Cytosolic Protein Extraction Kit (Beyotime, China), respectively, and the protein concentration was detected by the BCA method, followed by heating to denature the proteins. Equal amounts (20 μg) of proteins were electrophoresed on SDS-PAGE gels and transferred to PVDF membranes (Millipore, Bedford, MA, United States). The membranes were placed in 5% skimmed milk for 1 h on a rotary shaker to block non-specific binding sites and then incubated with a primary specific antibody at 4 °C overnight. On day 2, after washing three times (10 min/time) with 1× TBST buffer, anti-mouse or anti-rabbit HRP-labeled secondary antibodies were added and incubated at room temperature for 1 h. After washing again, immunoblotting analysis was performed using an ECL detection reagent (Beyotime, China). The primary antibodies used in this study were as follows: (Affinity, US) anti-Nrf2 (1:1000), anti-Bax (1:1000), anti-Bcl2 (1:1000), anti-HO-1 (1:1000), anti-NQO-1 (1:1000), anti-DCN (1:1000), anti-type Ⅱ collagen (1:1000), anti-MMP3 (1: 1000), anti-cleaved-caspase3 (1:1000) anti-LamⅡinB (1:1000), anti-βactin (1:1000), (abcom, US) anti-Bcl2 (1:1000) (CST, US) anti-cleaved-caspase3 (1:1000), (HUAAN, CN) anti-Nrf2 (1:1000).

### 2.5 qRT-PCR

Total cellular RNA was extracted using TRIzol (Invitrogen, United States), cDNA was synthesized by reverse transcription using The PrimeScript RT kit (Takara, Japan), and primers for Nrf2, HO-1, NQO-1, and β-actin were purchased from Reebok Bio. Real-time PCR was performed using the miDETECT A TrackTM miRNA qRT-PCR Starter Kit (Ribobio, China) for real-time PCR. β-actin was regarded as an endogenous control, and the relative mRNA expression was calculated using the 2^−ΔΔCt^ method. The primer sequences used were as Table 1.

**TABLE 1:** Sequence of primer.

Gene	Primer	NCBI No.
Nrf2	F: TCACACGAGATGAGCTTAGGGCAA	NM_010902.4
	R: TACAGTTCTGGGCGGCGACTTTAT	
HO-1	F: ATGGCCTCCCTGTACCACATC	NM_002133.2
	R: TGTTGCGCTCAATCTCCTCCT	
NQO-1	F: GGTGAGCTGAAGGACTCGAA	NM_008706.5
	R: ACCACTGCAATGGGAACTGAA	
β-actin	F: CAAGAGAGGTATCCTGACCT	NM_007393.5
	R: TGATCTGGGTCATCTTTTCAC	

### 2.6 Immunofluorescence

After drug treatment, NPC were inoculated on glass coverslips. They were then fixed with paraformaldehyde for 15 min at −20 °C, washed with PBS, and then membrane-broken with PBS containing 0.5% Triton X-100 for 30 min, washed with PBS, and then closed with 5% goat serum for 1 h at room temperature. The membranes were incubated with anti-Nrf2 (Affinity, US) at a dilution of 1:200 in blocking buffer overnight at 4 °C, washed with PBS, and incubated with fluorescent secondary antibody Flour-488 for 1 h at room temperature. Then, they visualized the changes in Nrf2 expression using a fluorescence microscope (Olympus, Japan).

### 2.7 Flow cytometer

Cells in each group were digested with 0.25% trypsin, washed, resuspended three times in pre-cooled PBS buffer, and stained using the Annexin V-FITC Apoptosis Detection Kit (Best boo, China) according to the manufacturer’s instructions. Annexin V-FITC (5 μL) was added and incubated for 15 min at room temperature away from light, and propidium iodide (PI) was added and incubated for 5 min away from sunlight. The apoptosis rate was detected using Cyto FLEX flow cytometry (Beckman, US).

### 2.8 Hurst staining

Cells were first pretreated with Ator for 6 h. Subsequently, cells were treated with 300 UmH_2_O_2_ in dishes with or without Ator for 6 h. PBS was rinsed three times, and Hoechst 33342 solution was added and incubated for 20–30 min. Coverslips were sealed, and morphologic changes in the nuclei of apoptotic cells were observed using a fluorescence microscope (Olympus, Japan).

### 2.9 SOD and MDA assay

Superoxide dismutase (SOD) and lipid oxidation (MDA) activities were analyzed using WST-8 Total Superoxide Dismutase Assay Kit (Beyotim, China) and Lipid Oxidation (MDA) Assay Kit (Beyotim, China), respectively, and the cell lysate and supernatant were added in 96-well plates according to the instruction manual, and the reaction was carried out with a microplate reader at 450 nm. The absorbance of each well was detected at 450 nm. And the total SOD and MDA contents were calculated according to the formula.

### 2.10 Animal experiments

Animal experiments were conducted using Sprague-Dawley (SD) rats (200 g ± 20 g, obtained from Jinan Pangyue Animal Experiment Center), and the College Ethics Committee of Economics and Medical College approved the animal experimental protocol (INRM-2023-DW-101). Fifteen two-month-old SD rats were randomly divided into three groups, namely, the control, acupuncture, and treatment groups, with three rats in each group. After anesthesia with sodium pentobarbital (40 mg/kg), the injection site was sterilized three times with iodine solution. Under a portable high-frequency X-ray device, a 25-gauge sterile needle punctured the AF layer, rotating the needle 360° and pumping it back slightly to create a model of IDD (Hu et al., 2018). Ator solution dissolved in DMSO was injected into the two IVDs closest to the caudal root using a 32G needle and a microsyringe. After 4 weeks, SD rats were executed by dragging the neck, and the disc tissues were peeled off. Tissue sections were prepared and stained with HE, FlipRed O, and immunohistochemical staining, and the morphology of the discs was observed under the microscope.

### 2.11 HE and flip red O staining

After 4 weeks of needling, the rats were decapitated, the whole tail was removed and fixed in fixative (Servicebio G1101) for 2 days, the vertebral bone was incised in the mid-sagittal plane, and the disc tissue was removed and dehydrated in a dehydrator, followed by embedding in paraffin wax. Then, the trimmed blocks were placed in paraffin slicers to make sections of 4 μm in thickness. The cells were deparaffinized, stained with hematoxylin and eosin (Servicebio G1003) and fuchsin O (Servicebio G1015), dehydrated and sealed, and then observed microscopically for the cells of the annulus fibrosus (AF) and the nucleus pulposus (NP). (After the sections were deparaffinized, stained with hematoxylin and eosin (Servicebio G1003) and saffron O (Servicebio G1015), dehydrated and sealed, the cytoarchitecture and morphology of the annulus fibrosus (AF) and the nucleus pulposus (NP) were observed using a microscope and the boundary between the two structures was evaluated.

### 2.12 Immunohistochemical staining

Paraffin sections were deparaffinized and incubated in 3% H_2_O_2_ solution at room temperature for 25 min, avoiding light, and washed 3 times with PBS for 5 min each time. 3% BSA was added to cover the tissue evenly, and the tissue was closed at room temperature for 30 min. Type Ⅱ collagen antibody (Servicebio 1:200) or MMP3 (Servicebio 1:200) was added dropwise and incubated overnight, then washed 3 times with PBS and added DAB color solution (Servicebio), and color development was controlled under the microscope. Wash with PBS for 3 times, add secondary antibody (Servicebio), and incubate for 50 min at room temperature; wash with PBS for 3 times, add DAB color development solution (Servicebio) drop by drop, and control the color development time under the microscope, the cheerful color is brownish-yellow, further hematoxylin re-staining after rinsing with tap water and then read the results under the white light microscope after sealing the film.

### 2.13 Statistical analysis

Data were analyzed using GraphPad Prism 8 software, and the results are the mean ± SD of three independent experiments. T-tests or one-way ANOVA were used to assess differences between group means. Differences were considered statistically significant at P < 0.05.

## 3 Results

### 3.1 Ator ameliorates H_2_O_2_ induced oxidative stress injury in NPC

**FIGURE 1:**
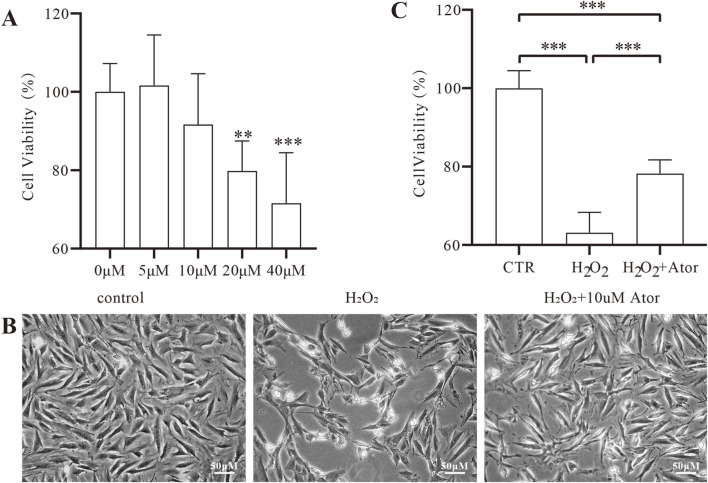
Ator ameliorates H_2_O_2_ induced oxidative stress injury in NPC. **(A)** Detection of drug toxicity by CCK-8 assay. The results are expressed as the mean ± standard deviation, n = 5 for each group. **(B)** Cell morphology under the microscope after pretreatment with 10 μM Ator for 6 h followed by exposure to 300 μM/mL H_2_O_2_ for 6 h. **(C)** Detection of cell viability of NPC by CCK-8 assay. All the data are shown as mean ± deviation, n = 6. **P < 0.01, ***P < 0.001. Abbreviations: CTR, control,; Ator, atorvastatin.

To determine the cytotoxicity of different concentrations of Ator and to select the optimal concentration, we treated NPC with different concentration gradients of Ator in the interval of 0–40 umol/mL. After incubation for 24 h, the cell viability of the other groups was observed using the CCK-8 kit. The results showed no significant change in cell viability in the Ator group in the 5–10 umol/mL concentration range (Figure 1A; Supplementary Table S1). To induce oxidative stress in NPC, we chose H_2_O_2_ as a stimulus to establish an *in vitro* IDD model. We set the concentration at 300 μmol/mL because cytotoxicity and morphological alterations could be induced in NPC using this concentration of H_2_O_2_ (Jia et al., 2017). After 12 h of treatment of NPC with 300umol/mL of H_2_O_2_, the morphology of NPC was significantly altered (Figure 1B; Supplementary Table S2): The cell outline was unclear, the total number of cells decreased, and the cell viability was reduced considerably (Figure 1C), but with Ator, the morphology of the cells was improved, the number of cells increased, and the cell viability was significantly enhanced. In summary, Ator improved H_2_O_2_-induced alterations in cell viability and morphology.

### 3.2 Ator enhances the antioxidant capacity of rat NPC

**FIGURE 2:**
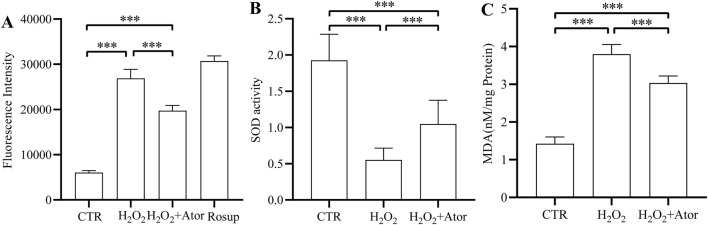
Ator enhance the antioxidant capacity of rat NPC. **(A)** Levels of ROS expression in NPC. **(B,C)** Levels of antioxidant enzymes SOD and MDA expression. All the data are shown as mean ± deviation, n = 6. ***P < 0.001. Abbreviations: CTR, control; Ator, atorvastatin; SOD, superoxide dismutase; MDA, malondialdehyde.

Subsequently, we examined the antioxidant capacity of myeloid cells after atorvastatin treatment, and we found that Atot significantly reduced the level of ROS after H_2_O_2_ treatment (Figure 2A; Supplementary Table S3). Superoxide dismutase plays an essential role in the regulation of oxidative stress (Wang et al., 2018). We found that adding H_2_O_2_ decreased SOD activation while adding Ator attenuated the inhibitory effect of H_2_O_2_ on SOD activity (Figure 2B; Supplementary Table S4). Lipid oxidation occurs when oxidative stress occurs, and malondialdehyde (MDA) is a breakdown product of lipid oxidation, which was found to increase after adding H_2_O_2_ and decreased after Ator treatment by testing MDA levels (Figure 2C; Supplementary Table S5). Therefore, the above results suggest that Ator reduced the accumulation of ROS in NPC and enhanced the antioxidant enzyme activity to enhance the antioxidant capacity of NPC.

### 3.3 Ator promotes the expression of Nrf2 and the activation of its downstream genes

**FIGURE 3:**
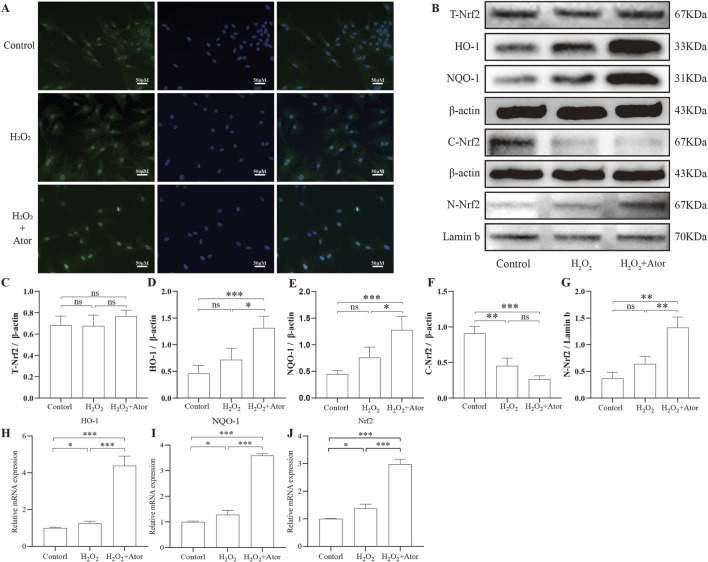
Ator promotes Nrf2 expression and its downstream gene activation. Nrf2 expression was detected by pretreatment with Ator for 6 h with the addition of 300 μM H_2_O_2_. **(A)** Immunofluorescence for Nrf2 expression. **(B–G)** Western blotting was performed to detect the expression of Total-Nrf2, HO-1, NQO-1, and Cytosolic-Nrf2 with β-actin as an internal reference and Nuclear-Nrf2 with Lamin-b as an internal reference. **(H–J)** Detection of Nrf2, NQO-1, and HO-1 expression using qPCR. All the data are shown as mean ± deviation, n = 3. *P < 0.05, **P < 0.01, ***P < 0.001. Abbreviations: Ator, atorvastatin; ns, non-significant.

First, we observed the intranuclear translocation of Nrf2 by immunofluorescence detection (Figure 3A). By analyzing the results of Western blotting, we found that the expression of Nrf2 in NPC was significantly increased in the nucleus and decreased in the cytoplasm after the addition of Ator, and the elevation of total Nrf2 expression was not obvious (Figures 3B–G; Supplementary Table S6), but we could see that the total Nrf2 expression was indeed elevated by PCR detection (Figure 3J). HO-1 and NQO-1 are downstream genes of Nrf2 that play a key role in resistance to oxidative stress. We examined the expression of these two genes and found that the mRNA expression of HO-1 and NQO-1 was significantly increased after the addition of Ator (Figure 3H,I; Supplementary Table S7). By Western blotting, we also obtained similar results, which confirmed the activation of the antioxidant function of Nrf2 (Figure 3B; Supplementary Table S6). Taken together, these results suggest that Ator enhances the expression of Nrf2 and promotes the expression of its downstream antioxidant genes, thereby exerting antioxidant effects in rat NPC.

### 3.4 Activation of Nrf2 is necessary for the antioxidant effect of Ator

**FIGURE 4:**
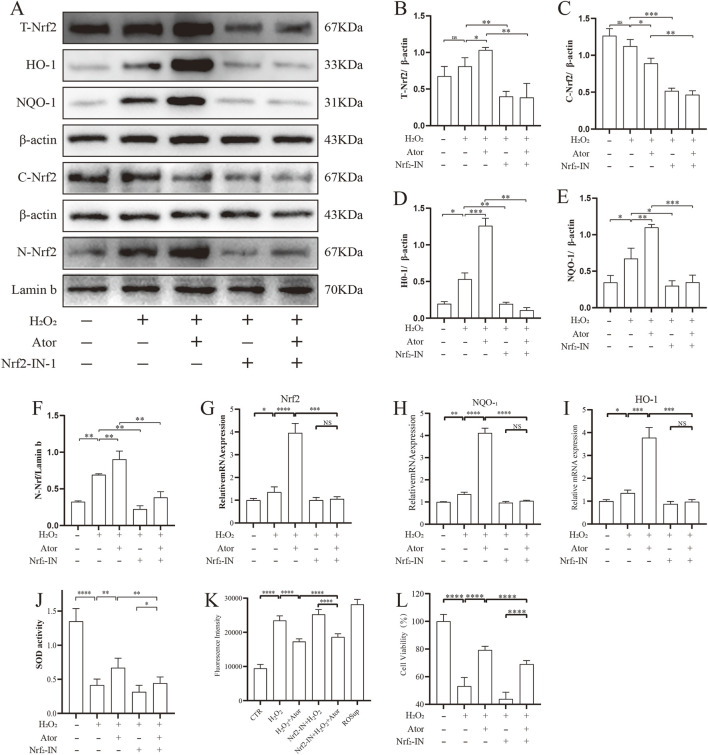
Activation of Nrf2 is necessary for Ator to exert antioxidant effects. Addition of Nrf2-IN-1 treatment followed by Ator pretreatment for 6 h and H_2_O_2_ treatment for 6 h **(A–F)** Western blotting for detection of relevant protein expression. Data are shown as mean ± deviation, n = 3. **(G–I)** qPCR for Nrf2, NQO-1, HO-1 expression. Data are shown as mean ± deviation, n = 3. **(J)** Antioxidant enzyme SOD expression. **(K)** ROS expression level. **(L)** CCK8 assay for cellular activity. Data are shown as mean ± deviation, n = 6. *P < 0.05, **P < 0.01, ***P < 0.001. Abbreviations: Ator, atorvastatin; Nrf2-IN-1, Nrf2 inhibitor; ns, non-significant.

To test the role of Nrf2 in the exertion of antioxidant function by Ator, we inhibited it using an inhibitor of Nrf2 (Nrf2-IN-1), and the addition of the Nrf2 inhibitor significantly inhibited the intranuclear translocation of Nrf2 and decreased the expression of its downstream associated proteins HO-1 and NQO-1 after Ator treatment compared to Ator-treated group (Figures 4A–F; Supplementary Table S8). Similar results were obtained by qPCR (Figure 4G–I, Supplementary Table S9), and the addition of the inhibitor reduced the level of SOD compared with the treatment group (Figure 4J; Supplementary Table S10). The Nrf2 inhibitor reduced the antioxidant effect of Ator by assaying the ROS level (Figure 4K; Supplementary Table S11). We measured cell viability, and the results showed that the addition of an Nrf2 inhibitor weakened the protective effect of Ator and decreased cell viability (Figure 4L; Supplementary Table S12). These results suggest that the activation of Nrf2 is necessary for the protective effect of Ator.

### 3.5 Ator slows NPC apoptosis

**FIGURE 5:**
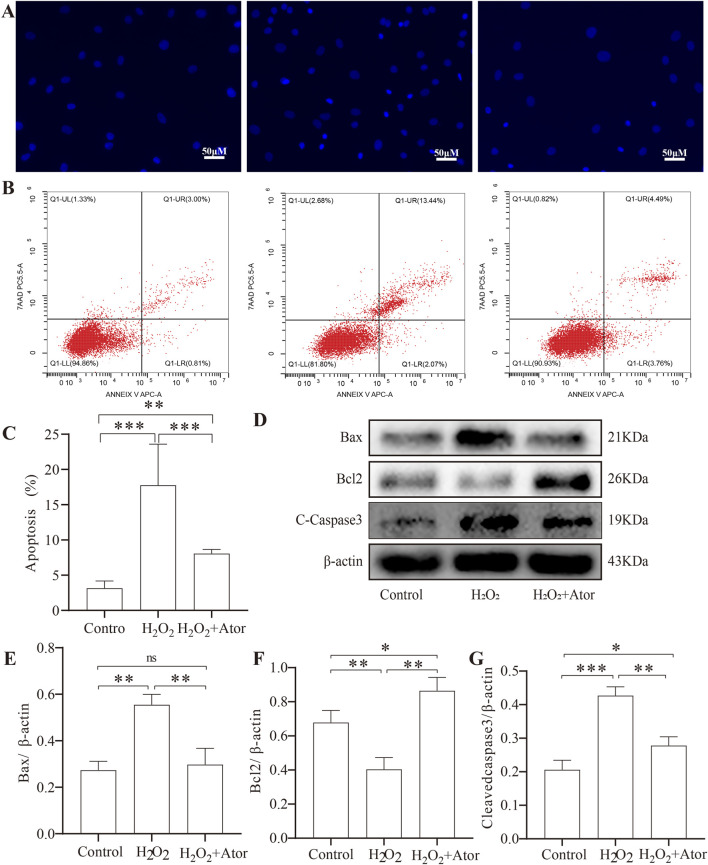
Ator slowed down apoptosis in NPC. Ator pretreated NPC for 6 h and added H_2_O_2_for 6 h. **(A)**Hurst staining for apoptosis. **(B,C)**Flow cytometry for NPC apoptosis and quantitative analysis. Data are shown as mean ± deviation, n = 3. **(D–G)**Western blotting for apoptosis-related protein expression. Data are shown as mean ± deviation, n = 3. *P < 0.05, **P < 0.01, ***P < 0.001. Abbreviations: Ator, atorvastatin; ns, non-significant.

We explored the effect of Ator on H_2_O_2_-induced apoptosis of NPCs. We pretreated the cells with 10 μM Ator for 6 h, followed by treatment with 300Π μM H_2_O_2_ for 6 h to induce apoptosis (Figures 5A–C). Western blotting results showed that the expression of apoptosis-related proteins cleaved-caspase3 and bax was increased, and bcl2 expression was decreased in the H_2_O_2_ group compared with the control group. The term of cleaved-caspase3 and bax was decreased, and bcl2 expression was elevated in the Ator-treated group compared with the H_2_O_2_ group (Figures 5D–G; Supplementary Table S13). To further confirm our hypothesis, we used Hochest staining and flow cytometry for the analysis. The results of Hochest staining showed a significant increase in the number of nuclear consolidated cells after H_2_O_2_ treatment. However, this percentage was significantly reduced when we added Ator for pretreatment (Figure 5E). The results of flow cytometry were also consistent with this (Figure 5F; Supplementary Figure S1; Supplementary Table S14). These findings suggest that Ator is effective in reducing H_2_O_2_-triggered apoptosis in NPC.

### 3.6 Intradiscal annotation of Ator delays needle-induced disc degeneration

**FIGURE 6:**
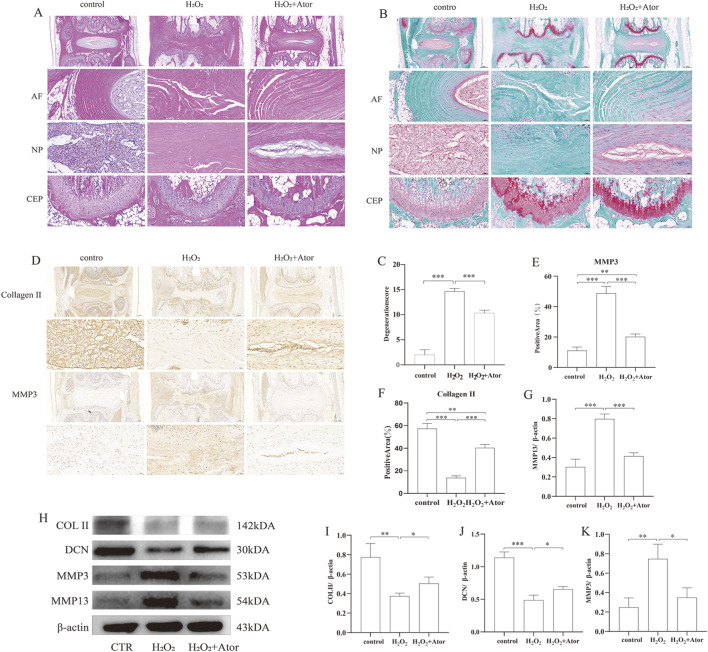
Intradiscal annotation of Ator delays needle-prick-induced IDD. The rat IDD model was constructed by needling, and Ator was injected into the rat intervertebral disc through a micro syringe at a concentration of Ator Four weeks later, the rats were executed to take the intervertebral discs for the next step of the experiments. **(A)** HE staining of rat intervertebral discs. **(B)** Flip-red O staining of rat intervertebral discs. **(C)** Standardized scoring scale of IDD in the rat was performed for scoring. Data are shown as mean ± deviation, n = 3. **(D–F)** Immunohistochemistry to detect the expression of Collagen Ⅱ and MMP3. Data are shown as mean ± deviation, n = 3. **(G–K)** Western blotting to detect the expression of NP tissue-associated proteins. Data are shown as mean ± deviation, n = 3. *P < 0.05, **P < 0.01, ***P < 0.001. Abbreviations: Ator, atorvastatin; AF, annulus fibrosus; NP, nucleus pulposus; CEP, cartilaginous endplate.

To study the effect of Ator on disc degeneration *in vivo*, we constructed a rat model of disc degeneration using the rat tail puncture method as the needling group. On this basis, some rats were treated with injections of Ator solution as the treatment group.HE staining showed that in the control group, the annulus fibrosus was intact and undamaged, with a clear border of the nucleus pulposus. In contrast, in the needling group, the annulus fibrosus was disrupted, and the nucleus pulposus disappeared and was replaced by fibrous tissue. However, adding Ator treatment improved the damage caused by needling (Figure 6A). Similar results were obtained with Turning Red O staining; Ator also enhanced the continuity of the upper and lower cartilage endplates (Figure 6B) and improved the quality of the discs according to the method established by Lai et al. (2021). Set a standardized rating scale for IDD in rats (Figure 6C). Next, we examined the protein expression of MMP3 as well as type Ⅱ collagen by immunohistochemistry, and needling caused an enhancement of MMP3 protein expression and a decrease in type Ⅱ collagen expression, which was reversed by Ator treatment (Figures 6D–F; Supplementary Table S15). We also isolated NP tissues from the lumbar intervertebral discs of adult rats for further study, and based on the results of Western blotting, it was clear that intravertebral disc injection of Ator effectively alleviated lumbar IDD caused by needling (Figures 6G–K; Supplementary Table S16). In summary, Ator helps maintain the height of the intervertebral space, protects the integrity of the annulus fibrosus, and effectively inhibits the onset of degeneration.

## 4 Discussion

Previous experiments preliminarily revealed that Ator ameliorates oxidative damage induced by H_2_O_2_ in NPC. We observed that Ator could reduce the accumulation of ROS and elevate the activity of antioxidant enzymes. More importantly, Ator was able to promote the activation of Nrf2 and drive its transfer from the cytoplasm to the nucleus, which in turn promoted the expression of its downstream antioxidant-associated proteins. This process significantly reduced the oxidative stress in medullary cells and minimized apoptosis. In addition, we found that Ator could reverse H_2_O_2_ induced extracellular matrix metabolic disturbances, including degradation of collagen type Ⅱ and core proteoglycan and increased expression of MMP3 and MMP13. To verify the effect of Ator on IDD *in vivo*, we constructed a rat IDD model using the acupuncture method. We injected Ator into its lumbar intervertebral discs, and the results showed that Ator was effective in delaying H_2_O_2_-induced IDD through observing H&E staining and sapphire red staining.

A growing body of research reveals the critical role of oxidative stress in IDD. During normal physiological processes, cells continuously produce active (Checa and Aran, 2020), and the balance between the oxidative and antioxidant systems in the body plays a crucial role in maintaining cellular homeostasis. When intracellular ROS production exceeds the threshold of natural antioxidant defense, oxidative stress occurs in the cell and leads to cell death (Xia et al., 2019; Belenguer-Varea et al., 2020). Excess accumulation of ROS plays an important role in many diseases such as atherosclerosis, neurodegenerative diseases, and IDD (Smith et al., 2016). High levels of oxidative stress can be detected in degenerating NPC, suggesting that oxidative stress is involved in NP degeneration. Many pathological factors, such as mechanical stress, high glucose, and H_2_O_2_, can induce oxidative stress in nucleus pulposus cells, and oxidative stress can cause mitochondrial damage and overproduction of reactive oxygen species, which can interfere with intervertebral disc matrix homeostasis by regulating matrix metabolism, proinflammation, apoptosis, and focal death (Yang and Lian, 2020; He et al., 2017; Harijith et al., 2014). Nrf2 protein expression plays an important role in combating oxidative stress. During the onset of oxidative stress, Nrf2 is activated and detached from Keap1 and enters the nucleus, enabling the expression of downstream antioxidant proteins that exert antioxidant effects (Zhang et al., 2023; Wang et al., 2023). The antioxidant role of Nrf2 is also emphasized. In cellular experiments, we chose H_2_O_2_ as an inducer of oxidative stress, and we found that Ator increased the aggregation of Nrf2 in the nucleus, significantly increased the expression of its downstream NQO-1 and HO-1 antioxidant proteins, and decreased ROS. However, as expected, adding an inhibitor of Nrf2 eliminated the antioxidant effect of Ator, which further reveals a novel mechanism of statins in treating IDD.

Apoptosis is a common type of programmed cell death. Its main histopathologic features include contraction of the cell membrane, blistering of the plasma membrane, condensation of the nucleus and cytoplasm, and generation of apoptotic vesicles. These apoptotic vesicles are subsequently phagocytosed by macrophages or neighboring cells but do not trigger an inflammatory response. The main apoptosis pathways include the death receptor, mitochondrial, and endoplasmic reticulum pathways (Bertheloot et al., 2021). Multiple mitochondrial ways can promote the formation of apoptotic complexes, including ROS generation. There is a close relationship between apoptosis and disc degeneration. It has been found that during disc degeneration, there is a large number of apoptotic cells in the intervertebral disc, especially those in the annulus fibrosus and nucleus pulposus (Yang et al., 2022). This apoptosis may be triggered by oxidative stress, inflammatory response, and mechanical stress within the cells. Apoptosis leads to a decrease in intervertebral disc cells and a decline in cell function, further accelerating the degenerative process of the intervertebral disc. Considering the relationship between oxidative stress and apoptosis, we examined the expression levels of apoptosis-related proteins. After the addition of H_2_O_2_, NPC underwent oxidative stress, which was detected by Western blotting and revealed that the decrease of Bcl2/Bax ratio and the increase of cleaved-caspase3 expression induced apoptosis, and this alteration was reversed by Ator, and this result was obtained by experiments such as flow cytometry and Hoechst staining, etc. This result was verified by flow cytometry and Hoechst staining, thus Ator can negatively regulate apoptosis through the Nrf2-HO-1 signaling pathway.

NPC and extracellular matrix form a major part of the NP structure and they play an important role in maintaining disc homeostasis (Kos et al., 2019). However, when the intervertebral disc degenerates, the extracellular matrix is disorganized, mainly manifested by the decrease in the activity of extracellular matrix synthesizing enzymes (Collagen Ⅱ, DCN), weakening the synthesis of new extracellular matrix. At the same time, the movement of extracellular matrix-degrading enzymes (MMP3, MMP13) increases, resulting in faster degradation of collagen and proteoglycans. This imbalance between synthesis and catabolism ultimately induces degradation of the extracellular matrix, causing degeneration (Li et al., 2022; Liang et al., 2022). Our *in vitro* experiments showed that Ator could reverse the extracellular matrix degradation induced by H_2_O_2_. Since disruption of the fibrous ring induces apoptosis and oxidative stress in NPC (Qian et al., 2019), we chose acupuncture to generate IDD, and compared with the acupuncture group alone, the height of the intervertebral space increased. The expression of collagen type Ⅱ and core proteoglycan increased. In contrast, the expression of MMP3 and MMP13 decreased, thus slowing down the degradation of the extracellular matrix and maintaining the stability of the intervertebral discs after adding Ator.

Despite the results of our study, there are some limitations. For example, we did not delve into the upstream mechanism of Nrf2-HO-1 signaling, while our study was limited to detecting apoptosis-related proteins without examining mitochondrial function. In addition, our study focused on exploring the effect of Nrf2 on apoptosis, but its impact on inflammation was not studied in depth. In future studies, we will improve and deepen these issues.

**FIGURE 7:**
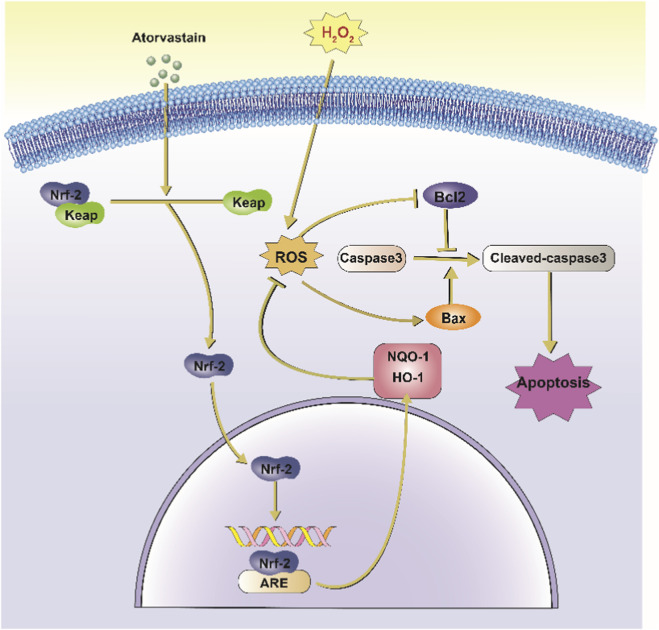
Schematic representation of Ator inhibition of H_2_O_2_-induced apoptosis in NPCs. Ator action on NPC causes Nrf2 to separate from Keap1 and enter the nucleus, which combines with ARE to promote the translation of downstream antioxidant proteins such as HO-1 and NQO-1 and inhibit H_2_O_2_-induced oxidative stress. It also reduced the activation of caspase-3 and inhibited apoptosis in NPC. Abbreviations:Nrf2, Nuclear factor erythroid 2-related factor 2; Keap1, Kelch-like ECH-associated protein 1; ROS, Reactive Oxygen Species; ARE, Antioxidant Response Element; HO-1, Heme Oxygenase-1; NQO-1, NAD(P) H Quinone Oxidoreductase 1; Bcl2, B-cell lymphoma 2; Bax, Bcl-2-associated X protein; H_2_O_2_, Hydrogen Peroxide.

In conclusion, this study found that activation of oxidative stress promotes apoptosis of NPCs, which induces the onset of IDD (Figure 7). Our study reveals the critical role of oxidative stress in disc degeneration and further demonstrates the potential value of targeting oxidative stress as a treatment for disc degeneration. Our analysis also showed that Ator could promote Nrf2 expression and enhance the antioxidant capacity of cells, thereby inhibiting the activation of apoptosis and providing a new therapeutic idea for treating disc degeneration.

## Data Availability

The original contributions presented in the study are included in the article/Supplementary Material, further inquiries can be directed to the corresponding authors.
