# Supplementary material for: Molecular insights into Atorvastatin’s role in delaying intervertebral disc degeneration
Source: Front Cell Dev Biol. 2025 Dec 11;13:1693951. doi: 10.3389/fcell.2025.1693951 (PMC12738912; doi:10.3389/fcell.2025.1693951)
Supplement: Supplementary file 1 [file DataSheet1.zip › supplementary/figure s1 legend.docx]

Figure S1. Apoptosis rate of NPC in each group. (A) Double negative control group. (B) 7-AAD control group. (C) Annexin-V control group.（D-F）) Control groups.（G-I）H_2_O_2_ groups（J-L）H_2_O_2_+Ator groups
