# Supplementary material for: Molecular insights into Atorvastatin’s role in delaying intervertebral disc degeneration
Source: Front Cell Dev Biol. 2025 Dec 11;13:1693951. doi: 10.3389/fcell.2025.1693951 (PMC12738912; doi:10.3389/fcell.2025.1693951)
Supplement: Supplementary file 1 [file DataSheet1.zip › supplementary/Supplementary tables.docx]

Table S1 OD value and value-added activity of NPC in each group

|  | Blanks | 0μM | 5μM | 10μM | 20μM | 40μM |
| --- | --- | --- | --- | --- | --- | --- |
| OD value | 0.2334 | 1.6366 | 1.8478 | 1.7767 | 1.5220 | 1.4260 |
|  | 0.2725 | 1.7856 | 1.7172 | 1.4644 | 1.5450 | 1.1295 |
|  | 0.2486 | 1.5634 | 1.8073 | 1.5625 | 1.3567 | 1.4132 |
|  | 0.2668 | 1.7881 | 1.5567 | 1.7697 | 1.3081 | 1.3730 |
|  | 0.2243 | 1.8495 | 1.7724 | 1.5370 | 1.4768 | 1.2391 |
|  | 0.2643 | 1.7544 | 1.7659 | 1.4817 | 1.3855 | 1.2190 |
| Cell viability (%) |  | 93.7843 | 115.0513 | 109.9843 | 91.8330 | 84.9914 |
|  |  | 103.8433 | 95.4795 | 78.7721 | 84.0989 | 56.6387 |
|  |  | 88.7779 | 118.5503 | 99.9315 | 84.2790 | 88.5762 |
|  |  | 103.9992 | 84.7893 | 98.7905 | 68.4480 | 72.7141 |
|  |  | 107.9652 | 95.2560 | 80.7716 | 77.0674 | 62.4415 |
|  |  | 101.6925 | 100.7718 | 81.6992 | 75.2433 | 64.0695 |
| Mean value |  | 100.0104 | 101.6497 | 91.6582 | 79.8053 | 71.5719 |
| Standard deviation |  | 7.23387 | 12.87812 | 12.95312 | 7.66224 | 12.9088 |
| P |  |  | 0.791262 | 0.197966 | 0.0012671 | 0.000832 |

Note: P value indicates comparison with the 0µM group.

Table S2 OD value and value-added activity of NPC in each group

|  | Blanks | CTR | H_2_O_2_ | H_2_O_2_+Ator |
| --- | --- | --- | --- | --- |
| OD value | 0.2123 | 1.7274 | 1.2536 | 1.3811 |
|  | 0.2445 | 1.8526 | 1.1691 | 1.4072 |
|  | 0.2381 | 1.8238 | 1.2847 | 1.4992 |
|  | 0.2101 | 1.8895 | 1.3204 | 1.5828 |
|  | 0.2008 | 1.8894 | 1.2919 | 1.5070 |
|  | 0.2305 | 1.7404 | 1.0741 | 1.4580 |
| Cell viability (%) |  | 94.2109 | 68.7281 | 77.1434 |
|  |  | 102.0368 | 57.4964 | 72.3027 |
|  |  | 100.2085 | 66.0024 | 79.5295 |
|  |  | 104.2846 | 66.1129 | 81.7375 |
|  |  | 104.2539 | 64.6157 | 77.3540 |
|  |  | 94.9623 | 55.8712 | 81.2968 |
| Mean value |  | 99.9928 | 63.1378 | 78.2273 |
| Standard deviation |  | 4.46179 | 5.19848 | 3.47785 |
| *P |  | 0.0000 |  |  |
| **P |  |  | 0.0000 |  |
| ***P |  |  |  | 0.0000 |

Note: *P values indicate CTR group comparison with H_2_O_2_ group; **P values indicate H_2_O_2_ group comparison with Atou+H_2_O_2_ group；***P values indicate H_2_O_2_+Ator group comparison with CTR. CTR means control; Ator means atorvastatin.

Table S3 Fluorescence intensity of each group of NPC

|  | CTR | H_2_O_2_ | H_2_O_2_+Ator | ROSup |
| --- | --- | --- | --- | --- |
| Fluorescenceintensity | 6292 | 28108 | 20957 | 31505 |
|  | 6068 | 27918 | 19681 | 31687 |
|  | 6774 | 28543 | 18145 | 28714 |
|  | 5864 | 28144 | 18686 | 29993 |
|  | 5516 | 24175 | 21064 | 31439 |
|  | 5853 | 24488 | 19885 | 30928 |
| Mean value | 6061.1667 | 27729.333 | 19736.333 | 30711 |
| Standard deviation | 433.79185 | 835.12626 | 1175.8768 | 1154.43649 |
| *P |  | 0.000 |  |  |
| **P |  |  | 0.000 |  |
| ***P |  |  |  | 0.000 |

Note: *P values indicate CTR group comparison with H_2_O_2_ group；**P values indicate H_2_O_2_ group comparison with Atou+ H_2_O_2_ group；***P values indicate H_2_O_2_+Ator group comparison with CTR. CTR means control.Ator means atorvastatin.

Table S4 SOD enzyme activity of each group of NPC

|  | Blank1 | Blank2 | Blank3 | CTR | H_2_O_2_ | H_2_O_2_+Ator |
| --- | --- | --- | --- | --- | --- | --- |
| A450 OD value | 1.7916 | 0.2110 | 0.0558 | 0.5124 | 0.9338 | 0.7266 |
|  | 1.5797 | 0.2074 | 0.0496 | 0.6006 | 0.8579 | 0.8787 |
|  | 1.7529 | 0.2143 | 0.0550 | 0.6563 | 1.0703 | 0.6913 |
|  | 1.7734 | 0.2154 | 0.0524 | 0.5706 | 1.0912 | 0.7894 |
|  | 1.6495 | 0.2167 | 0.0513 | 0.5122 | 1.0509 | 0.9055 |
|  | 1.6200 | 0.2150 | 0.0482 | 0.5274 | 1.0744 | 0.7242 |
| Blank 1 - Blank 2 - (experimental group - Blank 3) |  |  |  | 1.1240 | 0.7026 | 0.9098 |
|  |  |  |  | 0.8213 | 0.5640 | 0.5432 |
|  |  |  |  | 0.9373 | 0.5233 | 0.9023 |
|  |  |  |  | 1.0398 | 0.5192 | 0.8210 |
|  |  |  |  | 0.9719 | 0.4332 | 0.5786 |
|  |  |  |  | 0.9258 | 0.3788 | 0.7290 |
| Percentage inhibition = [(A blank control 1 - A blank control 2) - (A sample - A blank control 3)]/(A blank control 1 - A blank control 2) × 100% |  |  |  | 71.1122 | 44.4515 | 57.5604 |
|  |  |  |  | 59.8484 | 41.0989 | 39.5832 |
|  |  |  |  | 60.9190 | 34.0114 | 58.6442 |
|  |  |  |  | 66.7394 | 33.3248 | 52.6958 |
|  |  |  |  | 67.8322 | 30.2345 | 40.3825 |
|  |  |  |  | 65.8932 | 26.9609 | 51.8861 |
| SOD enzyme activity unit = inhibition% / (100% - inhibition%)units |  |  |  | 2.4617 | 0.8002 | 1.3563 |
|  |  |  |  | 1.4906 | 0.6978 | 0.6552 |
|  |  |  |  | 1.5588 | 0.5154 | 1.4180 |
|  |  |  |  | 2.0066 | 0.4998 | 1.1140 |
|  |  |  |  | 2.1087 | 0.4334 | 0.6774 |
|  |  |  |  | 1.9320 | 0.3691 | 1.0784 |
| Mean value |  |  |  | 1.9264 | 0.5526 | 1.0499 |
| Standard deviation |  |  |  | 0.3295 | 0.1497 | 0.2969 |
| *P |  |  |  | 0.000 |  |  |
| **P |  |  |  |  | 0.0074 |  |
| ***P |  |  |  |  |  | 0.0013 |

Note: *P values indicate CTR group comparison with H_2_O_2_ group；**P values indicate H_2_O_2_ group comparison with Ator+ H_2_O_2_ group；***P values indicate H_2_O_2_+Ator group comparison with CTR. CTR means control. Ator means atorvastatin.

Table S5 MDA level of each group of NPC

|  | standard | CTR | H_2_O_2_ | H_2_O_2_+Ator |
| --- | --- | --- | --- | --- |
| A532 OD value | 0.0512 | 0.0941 | 0.2059 | 0.1688 |
|  | 0.0641 | 0.0909 | 0.1953 | 0.1661 |
|  | 0.1154 | 0.1031 | 0.1802 | 0.1544 |
|  | 0.1982 | 0.1069 | 0.1943 | 0.1683 |
|  | 0.2819 | 0.0987 | 0.1831 | 0.1579 |
|  | 0.6841 | 0.1071 | 0.1848 | 0.1535 |
| concentration of MDA  （μM） |  | 3.7953 | 12.5984 | 9.6772 |
|  |  | 3.5433 | 11.7638 | 9.4646 |
|  |  | 4.5039 | 10.5748 | 8.5433 |
|  |  | 4.8031 | 11.6850 | 9.6378 |
|  |  | 4.1575 | 10.8031 | 8.8189 |
|  |  | 4.8189 | 10.9370 | 8.4724 |
| MDA level（nM/mg Protein） |  | 1.2651 | 4.1995 | 3.2257 |
|  |  | 1.1811 | 3.9213 | 3.1549 |
|  |  | 1.5013 | 3.5249 | 2.8478 |
|  |  | 1.6010 | 3.8950 | 3.2126 |
|  |  | 1.3858 | 3.6010 | 2.9396 |
|  |  | 1.6063 | 3.6457 | 2.8241 |
| Mean value |  | 1.4234 | 3.7979 | 3.0341 |
| Standard deviation |  | 0.1614 | 0.2318 | 0.1688 |
| *P |  |  | 0.0000 | 0.0001 |
| **P |  |  |  | 0.0001 |

Note: *P values indicate CTR group comparison with H_2_O_2_ group; **P values indicate H_2_O_2_ group comparison with Ator+ H_2_O_2_ group; CTR means control. Ator means atorvastatin.

Table S6 Analysis of grayscale values of each group of strips

|  | CTR | H_2_O_2_ | Ator+ H_2_O_2_ |
| --- | --- | --- | --- |
| C-Nrf2/β-actin | 0.925727846 | 0.33176551 | 0.213798311 |
|  | 0.801469162 | 0.488902458 | 0.269160935 |
|  | 1.000471544 | 0.539876674 | 0.310174906 |
| Mean value | 0.909222851 | 0.453514881 | 0.264378051 |
| Standard deviation | 0.08207638 | 0.088569274 | 0.039490666 |
| *P | 0.005936753 |  |  |
| **P |  | 0.050940847 |  |
| ***P |  |  | 0.000559331 |
| NQO-1-1/β-actin | 0.376257145 | 0.581319351 | 1.054470151 |
|  | 0.463914654 | 0.735089363 | 1.231503155 |
|  | 0.506270293 | 0.966163865 | 1.554960238 |
| Mean value | 0.448814031 | 0.760857526 | 1.280311181 |
| Standard deviation | 0.054141027 | 0.158165154 | 0.207218483 |
| *P | 0.057596856 |  |  |
| **P |  | 0.047922955 |  |
| ***P |  |  | 0.005361647 |
| T-Nrf2/β-actin | 0.639623004 | 0.578010332 | 0.793771094 |
|  | 0.782057269 | 0.779840639 | 0.807532203 |
|  | 0.626184564 | 0.673997494 | 0.702649945 |
| Mean value | 0.682621612 | 0.677282822 | 0.767984414 |
| Standard deviation | 0.070525339 | 0.082429619 | 0.046538777 |
| *P | 0.947853968 |  |  |
| **P |  | 0.246866014 |  |
| ***P |  |  | 0.226287617 |
| HO-1/β-actin | 0.352969456 | 0.513139617 | 1.077561241 |
|  | 0.631144299 | 0.942623382 | 1.37522234 |
|  | 0.40215723 | 0.702653494 | 1.495791396 |
| Mean value | 0.462090328 | 0.719472164 | 1.316191659 |
| Standard deviation | 0.121214117 | 0.175738872 | 0.17576989 |
| *P | 0.163398214 |  |  |
| **P |  | 0.027398521 |  |
| ***P |  |  | 0.004811692 |

Note: *P values indicate CTR group comparison with H_2_O_2_ group; **P values indicate H_2_O_2_ group comparison with Atou+ H_2_O_2_ group; ***P values indicate H_2_O_2_+Ator group comparison with CTR. CTR means control. Ator means atorvastatin.

Table S7 qPCR value in each group

|  | CTR | | | | H_2_O_2_ | | | | Ator+H_2_O_2_ | | |
| --- | --- | --- | --- | --- | --- | --- | --- | --- | --- | --- | --- |
|  | Nrf2 | HO-1 | NQO-1 | Nrf2 | | HO-1 | NQO-1 | Nrf2 | | HO-1 | NQO-1 |
| CT value | 23.2874 | 23.3301 | 23.3614 | 22.8829 | | 23.0233 | 23.1057 | 21.7024 | | 21.1453 | 21.5441 |
|  | 23.2803 | 23.2353 | 23.3089 | 22.8481 | | 23.0907 | 23.0142 | 21.8023 | | 21.3008 | 21.475 |
|  | 23.2795 | 23.2868 | 23.2809 | 22.9171 | | 22.9212 | 22.9212 | 21.7177 | | 21.1127 | 21.4839 |
| ∆CT value | 3.1896 | 3.2323 | 3.2636 | 2.8961 | | 3.0365 | 3.1189 | 1.5853 | | 1.0282 | 1.427 |
|  | 3.1665 | 3.1215 | 3.1951 | 2.5503 | | 2.7929 | 2.7164 | 1.6944 | | 1.1929 | 1.3671 |
|  | 3.1624 | 3.1697 | 3.1638 | 2.7158 | | 2.7199 | 2.7199 | 1.5215 | | 0.9165 | 1.2877 |
| ∆∆CT | 0.0168 | 0.0578 | 0.0561 | -0.2935 | | -0.1958 | -0.1447 | -1.6043 | | -2.2041 | -1.8366 |
|  | -0.0063 | -0.053 | -0.0124 | -0.6162 | | -0.3286 | -0.4787 | -1.4721 | | -1.9286 | -1.828 |
|  | -0.0104 | -0.0048 | -0.0437 | -0.4466 | | -0.4498 | -0.4439 | -1.6409 | | -2.2532 | -1.8761 |
| 2^(-∆∆CT) | 0.9884 | 0.9607 | 0.9619 | 1.2256 | | 1.1454 | 1.1055 | 3.0405 | | 4.6079 | 3.5717 |
|  | 1.0044 | 1.0374 | 1.0086 | 1.5328 | | 1.2558 | 1.3935 | 2.7743 | | 3.8069 | 3.5504 |
|  | 1.0073 | 1.0033 | 1.0308 | 1.3628 | | 1.3659 | 1.3603 | 3.1186 | | 4.7674 | 3.6708 |
| Mean value | 1 | 1.0005 | 1.0004 | 1.3738 | | 1.2557 | 1.2864 | 2.9778 | | 4.394 | 3.5976 |
| Standarddeviation | 0.0083 | 0.0314 | 0.0287 | 0.1257 | | 0.09 | 0.1286 | 0.1474 | | 0.4203 | 0.0525 |
| *P |  |  |  | 0.0137 | | 0.0193 | 0.0373 | 0 | | 0.0003 | 0 |
| **P |  |  |  |  | |  |  | 0.0003 | | 0.0005 | 0 |

Note: *P values indicate CTR group comparison with H_2_O_2_ group; **P values indicate H_2_O_2_ group comparison with Ator+ H_2_O_2_ group. CTR means control. Ator means atorvastatin.

Table S8 Analysis of grayscale values of each group of strips

|  | CTR | H_2_O_2_ | Ator+H_2_O_2_ | Nrf2-IN-1+H_2_O_2_ | Ator+Nrf2-IN-1+H_2_O_2_ |
| --- | --- | --- | --- | --- | --- |
| T-Nrf2/β-actin | 0.669050367 | 0.882280975 | 0.880628954 | 0.440176346 | 0.177437831 |
|  | 0.811303264 | 0.875111173 | 0.980075572 | 0.437720042 | 0.558986317 |
|  | 0.547949062 | 0.676042725 | 0.860688337 | 0.317503152 | 0.417118291 |
| Mean value | 0.676100898 | 0.811144958 | 0.907130954 | 0.398466513 | 0.384514146 |
| Standard deviation | 0.10762943 | 0.095576537 | 0.0522181 | 0.057258523 | 0.157463402 |
| *P |  | 0.255248785 |  |  |  |
| **P |  |  | 0.280631956 |  |  |
| ***P |  |  |  | 0.006348098 |  |
| ****P |  |  |  |  | 0.01120208 |
| HO-1/β-actin | 0.184430339 | 0.589957817 | 1.514048876 | 0.206390869 | 0.072239791 |
|  | 0.228004073 | 0.571785485 | 1.535843366 | 0.207853798 | 0.140419861 |
|  | 0.173963825 | 0.435689266 | 1.274757656 | 0.169685446 | 0.117287206 |
| Mean value | 0.195466079 | 0.532477523 | 1.441549966 | 0.194643371 | 0.109982286 |
| Standard deviation | 0.02340125 | 0.068840557 | 0.11827512 | 0.017658021 | 0.02830962 |
| *P |  | 0.002801066 |  |  |  |
| **P |  |  | 0.00071543 |  |  |
| ***P |  |  |  | 0.002549805 |  |
| ****P |  |  |  |  | 0.000101536 |
| NQO-1/β-actin | 0.36933333 | 0.800031552 | 1.121428674 | 0.341251539 | 0.281982264 |
|  | 0.427537147 | 0.699914122 | 1.139344788 | 0.33970584 | 0.459940281 |
|  | 0.242556846 | 0.520291076 | 1.126041632 | 0.220596002 | 0.301113275 |
| Mean value | 0.346475774 | 0.67341225 | 1.128938365 | 0.300517794 | 0.347678607 |
| Standard deviation | 0.066881533 | 0.100225858 | 0.006577997 | 0.048944953 | 0.069077897 |
|  |  |  |  |  |  |
| *P |  | 0.029292198 |  |  |  |
| **P |  |  | 0.005141666 |  |  |
| ***P |  |  |  | 0.01491935 |  |
| ****P |  |  |  |  | 0.000160291 |
| C-Nrf2/β-actin | 1.3115424 | 1.215877563 | 1.051981461 | 0.394677932 | 0.206434876 |
|  | 1.53945657 | 1.391687584 | 1.123096051 | 0.417714275 | 0.376804359 |
|  | 1.319897445 | 1.213276699 | 0.819216307 | 0.434541272 | 0.395423819 |
| Mean value | 1.390298805 | 1.273613949 | 0.998097939 | 0.415644493 | 0.326221018 |
| Standard deviation | 0.105525608 | 0.083497419 | 0.129777502 | 0.016339818 | 0.085041993 |
| *P |  | 0.287334822 |  |  |  |
| **P |  |  | 0.065016058 |  |  |
| ***P |  |  |  | 0.000140422 |  |
| ****P |  |  |  |  | 0.00360187 |
| N-Nrf2/β-actin | 0.317813134 | 0.691102788 | 0.857466097 | 0.197244247 | 0.321610519 |
|  | 0.337971825 | 0.677986475 | 1.030895844 | 0.196525562 | 0.470562135 |
|  | 0.314525162 | 0.705522166 | 0.821682162 | 0.276948909 | 0.353159033 |
| Mean value | 0.323436707 | 0.691537143 | 0.903348034 | 0.223572906 | 0.381777229 |
| Standard deviation | 0.010365164 | 0.011245594 | 0.091365403 | 0.037743674 | 0.064087944 |
| *P |  | 0.001644168 |  |  |  |
| **P |  |  | 0.031257607 |  |  |
| ***P |  |  |  | 0.001824847 |  |
| ****P |  |  |  |  | 0.002716343 |

Note: *P values indicate CTR group comparison with H_2_O_2_ group; **P values indicate H_2_O_2_ group comparison with Ator+ H_2_O_2_ group; ***P values indicate H_2_O_2_ group comparison with H_2_O_2_+ Nrf2-IN-1 group; ****P values indicate Ator+ H_2_O_2_ group comparison with Ator+Nrf2-IN-1+ H_2_O_2_ group.

Table S9-1 qPCR value in each group

|  | Contorl | | | H_2_O_2_ | | | H_2_O_2_+Ator | | |
| --- | --- | --- | --- | --- | --- | --- | --- | --- | --- |
|  | Nrf2 | HO-1 | NQO-1 | Nrf2 | HO-1 | NQO-1 | Nrf2 | HO-1 | NQO-1 |
| CT value | 23.0520 | 22.9205 | 23.0917 | 22.6577 | 22.4695 | 22.5337 | 21.0873 | 21.0503 | 20.8948 |
|  | 23.1210 | 22.9867 | 22.9515 | 22.5477 | 22.5214 | 22.6155 | 21.0848 | 21.0080 | 21.0942 |
|  | 23.0659 | 22.9805 | 23.0681 | 22.5817 | 22.5117 | 22.5820 | 21.1010 | 21.0712 | 20.9767 |
| ∆CT value | 2.9045 | 2.7730 | 2.9442 | 2.5514 | 2.3632 | 2.4274 | 1.0766 | 1.0396 | 0.8841 |
|  | 3.0946 | 2.9603 | 2.9251 | 2.4045 | 2.3782 | 2.4723 | 0.9611 | 0.8843 | 0.9705 |
|  | 2.9024 | 2.8170 | 2.9046 | 2.5715 | 2.5015 | 2.5718 | 0.9246 | 0.8948 | 0.8003 |
| ∆∆CT | -0.0627 | -0.0771 | 0.0196 | -0.3531 | -0.4098 | -0.5168 | -1.8279 | -1.7334 | -2.0601 |
|  | 0.1274 | 0.1102 | 0.0005 | -0.6901 | -0.5821 | -0.4528 | -2.1335 | -2.0760 | -1.9546 |
|  | -0.0648 | -0.0331 | -0.0200 | -0.3309 | -0.3155 | -0.3328 | -1.9778 | -1.9222 | -2.1043 |
| 2^(-∆∆CT) | 1.0444 | 1.0549 | 0.9865 | 1.2773 | 1.3285 | 1.4308 | 3.5502 | 3.3251 | 4.1702 |
|  | 0.9155 | 0.9265 | 0.9997 | 1.6134 | 1.4970 | 1.3687 | 4.3878 | 4.2164 | 3.8761 |
|  | 1.0459 | 1.0232 | 1.0140 | 1.2578 | 1.2444 | 1.2595 | 3.9389 | 3.7900 | 4.2999 |
| Mean value | 1.0019 | 1.0015 | 1.0001 | 1.3828 | 1.3567 | 1.3530 | 3.9590 | 3.7772 | 4.1154 |
| Standard deviation | 0.0611 | 0.0546 | 0.0112 | 0.1632 | 0.1050 | 0.0708 | 0.3422 | 0.3640 | 0.1773 |
| *P |  |  |  | 0.036 | 0.013 | 0.002 | 0.000 | 0.0004 | 0.000 |
| **P |  |  |  |  |  |  | 0.000 | 0.0008 | 0.000 |

Note: *P values indicate CTR group comparison with H_2_O_2_ group; **P values indicate H_2_O_2_ group comparison with Ator+ H_2_O_2_ group.

Table S9-2 qPCR value in each group

|  | Nrf2-IN+H2O2 | | | Nrf2-IN+H2O2+Ator | | |
| --- | --- | --- | --- | --- | --- | --- |
|  | Nrf2 | HO-1 | NQO-1 | Nrf2 | HO-1 | NQO-1 |
| CT value | 23.0643 | 23.1224 | 22.9678 | 23.0240 | 23.0263 | 23.0042 |
|  | 23.0201 | 23.0626 | 23.1146 | 23.0459 | 23.0140 | 23.0558 |
|  | 23.0432 | 23.1795 | 23.0542 | 23.1436 | 23.1785 | 23.0273 |
| ∆CT value | 2.9882 | 3.0463 | 2.8917 | 2.8610 | 2.8633 | 2.8412 |
|  | 2.9098 | 2.9523 | 3.0043 | 2.8746 | 2.8427 | 2.8845 |
|  | 3.0045 | 3.1408 | 3.0155 | 2.9439 | 2.9788 | 2.8276 |
| ∆∆CT | 0.0837 | 0.2733 | -0.0525 | -0.0435 | 0.0903 | -0.1030 |
|  | -0.1848 | -0.0080 | 0.0792 | -0.2200 | -0.1176 | -0.0406 |
|  | 0.1021 | 0.3238 | 0.1109 | 0.0415 | 0.1618 | -0.0770 |
| 2^(-∆∆CT) | 0.9436 | 0.8274 | 1.0371 | 1.0306 | 0.9393 | 1.0740 |
|  | 1.1367 | 1.0056 | 0.9466 | 1.1647 | 1.0849 | 1.0285 |
|  | 0.9317 | 0.7990 | 0.9260 | 0.9716 | 0.8939 | 1.0548 |
| Mean value | 1.0040 | 0.8773 | 0.9699 | 1.0557 | 0.9727 | 1.0525 |
| Standard deviation | 0.0939 | 0.0914 | 0.0482 | 0.0808 | 0.0815 | 0.0186 |
| ***P |  |  |  | 0.000 | 0.000 | 0.000 |
| ****P |  |  |  | 0.587 | 0.332 | 0.086 |

Note: ***P values indicate H_2_O_2_ group comparison with H_2_O_2_+ Nrf2-IN-1 group; ****P values indicate Ator+ H_2_O_2_ group comparison with Ator+Nrf2-IN-1+ H_2_O_2_ group.

Table S10 SOD enzyme activity of each group of NPC

|  | Blank1 | Blank2 | Blank3 | CTR | H_2_O_2_ | H_2_O_2_+Ator | Nrf2-IN+H_2_O_2_ | Nrf2-IN+H_2_O_2_+Ator |
| --- | --- | --- | --- | --- | --- | --- | --- | --- |
| A450 OD value | 1.8164 | 0.1967 | 0.0445 | 0.7853 | 1.2334 | 1.1028 | 1.4986 | 1.2556 |
|  | 1.9625 | 0.1899 | 0.0470 | 0.9269 | 1.3516 | 1.1329 | 1.5390 | 1.3722 |
|  | 1.9806 | 0.1976 | 0.0592 | 0.7811 | 1.4183 | 1.2928 | 1.3872 | 1.3306 |
|  | 1.8253 | 0.2005 | 0.0589 | 0.8637 | 1.4803 | 1.1168 | 1.5231 | 1.2665 |
|  | 1.8122 | 0.1861 | 0.0576 | 0.8444 | 1.2993 | 1.2413 | 1.3137 | 1.4419 |
|  | 1.9328 | 0.1848 | 0.0532 | 0.8208 | 1.3440 | 1.0569 | 1.4545 | 1.2998 |
| Blank 1 - Blank 2 - (experimental group - Blank 3) |  |  |  | 1.0311 | 0.5830 | 0.7136 | 0.3178 | 0.5608 |
|  |  |  |  | 1.0356 | 0.6109 | 0.8296 | 0.4235 | 0.5903 |
|  |  |  |  | 1.1995 | 0.5623 | 0.6878 | 0.5934 | 0.6500 |
|  |  |  |  | 0.9616 | 0.3450 | 0.7085 | 0.3022 | 0.5588 |
|  |  |  |  | 0.9678 | 0.5129 | 0.5709 | 0.4985 | 0.3703 |
|  |  |  |  | 1.1120 | 0.5888 | 0.8759 | 0.4783 | 0.6330 |
| Percentage inhibition = [(A blank control 1 - A blank control 2) - (A sample - A blank control 3)]/(A blank control 1 - A blank control 2) × 100% |  |  |  | 58.19 | 32.90 | 40.27 | 17.94 | 31.65 |
|  |  |  |  | 54.06 | 31.89 | 43.31 | 22.11 | 30.82 |
|  |  |  |  | 62.43 | 29.27 | 35.80 | 30.88 | 33.83 |
|  |  |  |  | 54.44 | 19.53 | 40.11 | 17.11 | 31.63 |
|  |  |  |  | 55.16 | 29.23 | 32.54 | 28.41 | 21.10 |
|  |  |  |  | 59.16 | 31.33 | 46.60 | 25.45 | 33.68 |
| SOD enzyme activity unit = inhibition% /(100% - inhibition%)units |  |  |  | 1.3919 | 0.4904 | 0.6743 | 0.2186 | 0.4631 |
|  |  |  |  | 1.1770 | 0.4683 | 0.7640 | 0.2838 | 0.4454 |
|  |  |  |  | 1.6616 | 0.4137 | 0.5576 | 0.4468 | 0.5112 |
|  |  |  |  | 1.1948 | 0.2427 | 0.6697 | 0.2064 | 0.4627 |
|  |  |  |  | 1.2300 | 0.4131 | 0.4823 | 0.3969 | 0.2675 |
|  |  |  |  | 1.4487 | 0.4562 | 0.8727 | 0.3413 | 0.5078 |
| Mean value |  |  |  | 1.3507 | 0.4140 | 0.6701 | 0.3156 | 0.4430 |
| Standard deviation |  |  |  | 0.1717 | 0.0815 | 0.1277 | 0.0884 | 0.0821 |
| *P |  |  |  |  | 0.0000 |  |  |  |
| **P |  |  |  |  |  | 0.0036 |  |  |
| ***P |  |  |  |  |  |  |  | 0.0399 |
| ****P |  |  |  |  |  |  |  | 0.0074 |

Note: *P values indicate CTR group comparison with H_2_O_2_ group; **P values indicate H_2_O_2_ group comparison with Ator+H_2_O_2_ group; ***P values indicate H_2_O_2_ group comparison with H_2_O_2_+ Nrf2-IN-1 group; ****P values indicate Ator+H_2_O_2_ group comparison with Ator+Nrf2-IN-1+H_2_O_2_ group.

Table S11 Fluorescence intensity of each group of NPC

|  | CTR | H_2_O_2_ | H_2_O_2_+Ator | Nrf2-IN+H_2_O_2_ | Nrf2-IN+H_2_O_2_+Ator | ROSup |
| --- | --- | --- | --- | --- | --- | --- |
| Fluorescence intensity | 9291 | 25960 | 16367 | 23764 | 17506 | 29585 |
|  | 8027 | 23546 | 18016 | 24126 | 19078 | 26324 |
|  | 11019 | 23210 | 18058 | 26668 | 19835 | 26742 |
|  | 8436 | 23523 | 17469 | 26997 | 17636 | 29912 |
|  | 10357 | 22309 | 17629 | 24288 | 19373 | 28201 |
|  | 9647 | 22284 | 16214 | 25727 | 18468 | 28384 |
| Mean value | 9462.8333 | 23472 | 17292.16667 | 25261.66667 | 18649.33333 | 28191.333 |
| Standard deviation | 1032.8469 | 1226.8373 | 738.6405117 | 1270.594437 | 864.1172117 | 1324.6204 |
| *P | 0.000 |  |  |  |  |  |
| **P |  | 0.000 |  |  |  |  |
| ***P |  |  | 0.000 |  |  |  |
| ****P |  |  |  | 0.000 |  |  |

Note: *P values indicate CTR group comparison with H_2_O_2_ group; **P values indicate H_2_O_2_ group comparison with Ator+ H_2_O_2_ group; ***P values indicate H_2_O_2_ group comparison with H_2_O_2_+ Nrf2-IN-1 group; ****P values indicate Ator+ H_2_O_2_ group comparison with Ator+Nrf2-IN-1+ H_2_O_2_ group.

Table S12 OD value and value-added activity of NPC in each group

|  | Blake | CTR | H_2_O_2_ | H_2_O_2_+Ator | Nrf2-IN+H_2_O_2_ | Nrf2-IN+H_2_O_2_+Ator |
| --- | --- | --- | --- | --- | --- | --- |
| OD value | 0.1994 | 1.9694 | 1.0237 | 1.6356 | 1.0427 | 1.4045 |
|  | 0.2144 | 2.0469 | 1.0586 | 1.7132 | 1.0681 | 1.4169 |
|  | 0.2187 | 2.1616 | 1.2975 | 1.6998 | 1.0746 | 1.5496 |
|  | 0.2161 | 1.9612 | 1.2322 | 1.6507 | 1.0713 | 1.4686 |
|  | 0.2097 | 2.1275 | 1.1974 | 1.6840 | 0.9151 | 1.5201 |
|  | 0.2090 | 1.9456 | 1.2730 | 1.5568 | 0.8938 | 1.4653 |
| Cell viability (%) |  | 96.4052 | 46.5706 | 81.1412 | 47.6441 | 68.0847 |
|  |  | 100.6315 | 46.0682 | 81.7899 | 46.5866 | 65.6207 |
|  |  | 106.9467 | 55.5252 | 76.2314 | 44.0527 | 68.5007 |
|  |  | 95.9215 | 58.2259 | 82.2073 | 49.0058 | 71.7724 |
|  |  | 105.0446 | 51.5017 | 76.8745 | 36.7817 | 68.3283 |
|  |  | 95.0832 | 61.2691 | 77.6114 | 39.4334 | 72.3425 |
| Mean value |  | 100.0055 | 53.1935 | 79.3093 | 43.9174 | 69.1082 |
| Standard deviation |  | 4.6158 | 5.6785 | 2.4560 | 4.4337 | 2.3002 |
| *P |  |  | 0.0000 |  |  |  |
| **P |  |  |  | 0.0000 |  |  |
| ***P |  |  |  |  | 0.0000 |  |
| ****P |  |  |  |  |  | 0.0000 |

Note: *P values indicate CTR group comparison with H_2_O_2_ group; **P values indicate H_2_O_2_ group comparison with Ator+ H_2_O_2_ group; ***P values indicate H_2_O_2_ group comparison with H_2_O_2_+ Nrf2-IN-1 group; ****P values indicate Ator+ H_2_O_2_ group comparison with Ator+Nrf2-IN-1+ H_2_O_2_ group.

Table S13 Analysis of grayscale values of each group of strips

|  | CTR | H_2_O_2_ | H_2_O_2_+Ator |
| --- | --- | --- | --- |
| Bax/β-actin | 0.313788093 | 0.600111052 | 0.244685397 |
|  | 0.237420868 | 0.511513628 | 0.272084251 |
|  | 0.269657077 | 0.554283511 | 0.377173896 |
| Mean value | 0.273622013 | 0.55530273 | 0.297981181 |
| Standard deviation | 0.031302596 | 0.036176926 | 0.057103934 |
| *P | 0.001136494 |  |  |
| **P |  | 0.005755838 |  |
| ***P |  |  | 0.624798108 |
| Bcl2/β-actin | 0.610595246 | 0.409549841 | 0.941943557 |
|  | 0.675244516 | 0.332726929 | 0.867035189 |
|  | 0.751596702 | 0.469841336 | 0.785310211 |
| Mean value | 0.679145488 | 0.404039369 | 0.864762986 |
| Standard deviation | 0.057629656 | 0.056112174 | 0.063965478 |
| *P | 0.008418704 |  |  |
| **P |  | 0.001563124 |  |
| ***P |  |  | 0.038068961 |
| C-Caspase3/β-actin | 0.236947634 | 0.449377289 | 0.29588658 |
|  | 0.181109277 | 0.398567693 | 0.247858427 |
|  | 0.200510795 | 0.433031843 | 0.29049535 |
| Mean value | 0.206189235 | 0.426992275 | 0.278080119 |
| Standard deviation | 0.023146836 | 0.021177993 | 0.021483006 |
| *P | 0.000572316 |  |  |
| **P |  | 0.002214561 |  |

Note: *P values indicate H_2_O_2_ group comparison with CTR group; **P values indicate H_2_O_2_ group comparison with Ator+H_2_O_2_ group.

| Table S14 Apoptosis rate assay | | | |
| --- | --- | --- | --- |
|  | CTR | H2O2 | H2O2+Ator |
|  | 3.81 | 13.35 | 8.25 |
|  | 1.97 | 16.11 | 7.9 |
|  | 3.68 | 15.51 | 7.4 |
| Mean value | 3.1533 | 14.9900 | 7.8500 |
| Standard deviation | 0.8384 | 1.1852 | 0.3488 |
| *P |  | 0.0003 | 0.0019 |
| **P |  |  | 0.0012 |
| Note: *P values indicate H_2_O_2_ group comparison with CTR group; **P values indicate H_2_O_2_ group comparison with Ator+H_2_O_2_ group. | | | |


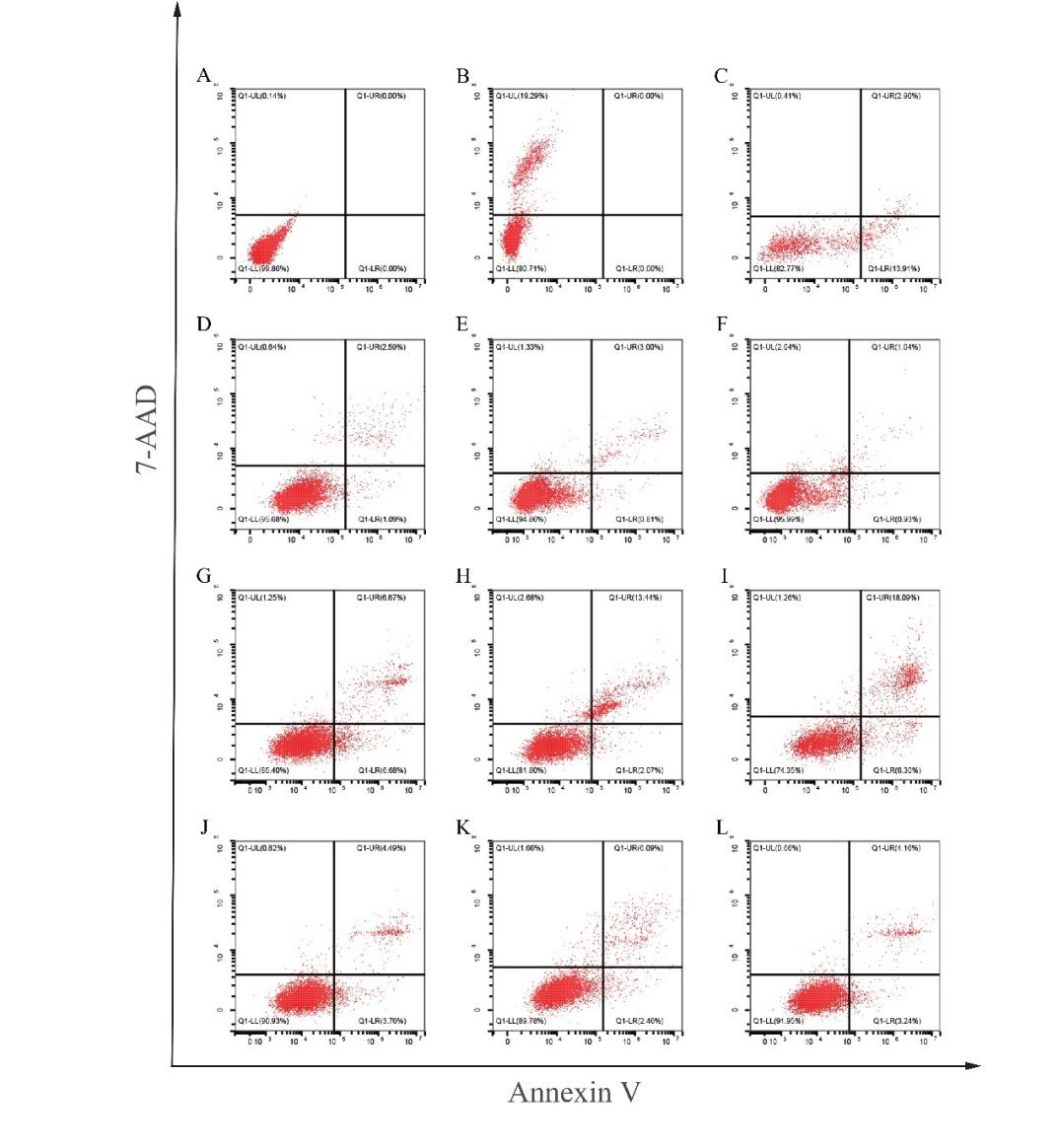


Figure S1. Apoptosis rate of NPC in each group. (A) Double negative control group. (B) 7-AAD control group. (C) Annexin-V control group.（D-F）) Control groups.（G-I）H_2_O_2_ groups（J-L）H_2_O_2_+Ator groups

Table S15 Immunohistochemical analysis of various groups of intervertebral discs

|  | CTR | H_2_O_2_ | H_2_O_2_+Ator | |
| --- | --- | --- | --- | --- |
| Collagen Ⅱ（mean） | 210.374 | 35.401 | 106.861 | |
|  | 201.445 | 33.215 | 97.661 | |
|  | 197.981 | 29.421 | 99.997 | |
| *P | 0.000165486 |  |  | |
| **P |  | 0.001025218 | |  |
| ***P |  |  | 0.000344702 | |
| MMP3(mean) | 7.886 | 144.516 | 12.903 | |
|  | 12.561 | 127.924 | 15.831 | |
|  | 9.773 | 157.973 | 27.663 | |
| *P | 0.005156138 |  |  | |
| **P |  | 0.002544397 | |  |
| ***P |  |  | 0.198901491 | |

Note: *P values indicate CTR group comparison with H_2_O_2_ group; **P values indicate H_2_O_2_ group comparison with Atou+H_2_O_2_ group; ***P values indicate H_2_O_2_+Ator group comparison with CTR. CTR means control. Ator means atorvastatin.

Table S16 Analysis of grayscale values of each group of strips

|  | CTR | H_2_O_2_ | Ator |
| --- | --- | --- | --- |
| Collagen Ⅱ/β-actin | 0.61441613 | 0.342483917 | 0.434725144 |
|  | 0.853014839 | 0.401780683 | 0.562608671 |
|  | 0.859836532 | 0.379085773 | 0.517561644 |
| Mean value | 0.775755833 | 0.374450124 | 0.504965153 |
| Standard deviation | 0.114118385 | 0.02442872 | 0.052962583 |
| *P | 0.008261276 |  |  |
| **P |  |  | 0.034032937 |
| DCN/β-actin | 1.07469714 | 0.401119004 | 0.609775279 |
|  | 1.238993636 | 0.525721663 | 0.681400397 |
|  | 1.10786682 | 0.53586938 | 0.673771916 |
| Mean value | 1.140519199 | 0.487570016 | 0.654982531 |
| Standard deviation | 0.070936441 | 0.061270314 | 0.032117701 |
| *P | 0.000595525 |  |  |
| **P |  |  | 0.026720465 |
| MMP 3/β-actin | 0.245872193 | 0.618801762 | 0.286575832 |
|  | 0.345935812 | 0.912724279 | 0.46372502 |
|  | 0.154892503 | 0.711000584 | 0.298359452 |
| Mean value | 0.248900169 | 0.747508875 | 0.349553435 |
| Standard deviation | 0.078022488 | 0.122738891 | 0.080874704 |
| *P | 0.008349282 |  |  |
| **P |  |  | 0.018636664 |
| MMP 13/β-actin | 0.356859541 | 0.771100247 | 0.417681512 |
|  | 0.3428622 | 0.855524281 | 0.447529078 |
|  | 0.211524262 | 0.766457568 | 0.379601443 |
| Mean value | 0.303748668 | 0.797694032 | 0.414937344 |
| Standard deviation | 0.065462392 | 0.040936063 | 0.027799146 |
| *P | 0.000826912 |  |  |
| **P |  |  | 0.000396646 |

Note: *P values indicate CTR group comparison with H_2_O_2_ group; **P values indicate H_2_O_2_ group comparison with Ator+H_2_O_2_ group. CTR means control. Ator means atorvastatin.
