# Supplementary figures and images for: Molecular insights into Atorvastatin’s role in delaying intervertebral disc degeneration
Source: Front Cell Dev Biol. 2025 Dec 11;13:1693951. doi: 10.3389/fcell.2025.1693951 (PMC12738912; doi:10.3389/fcell.2025.1693951)

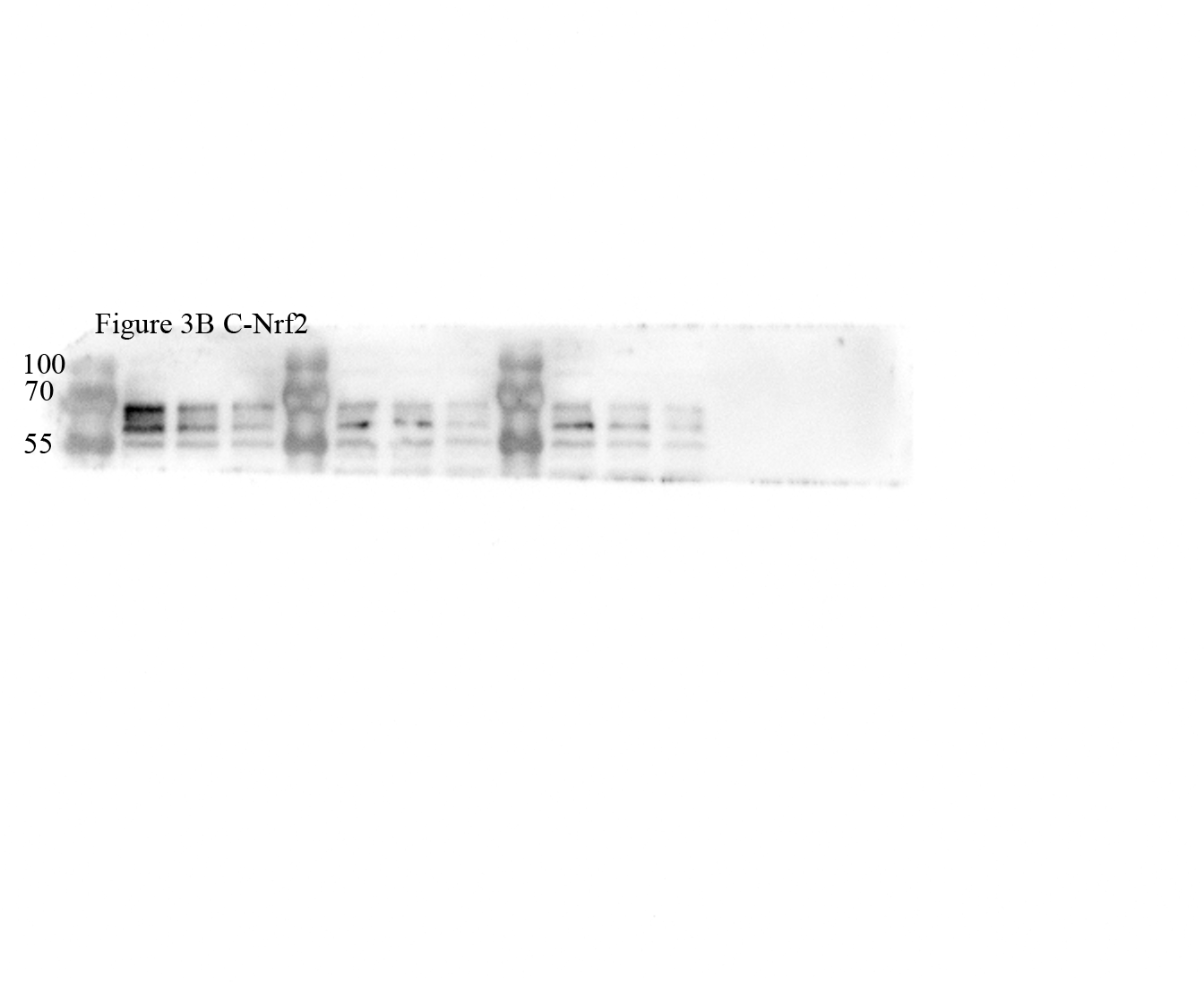

Supplement: Supplementary file 1 [file DataSheet1.zip › supplementary/Figure 3B C-Nrf2.tif]

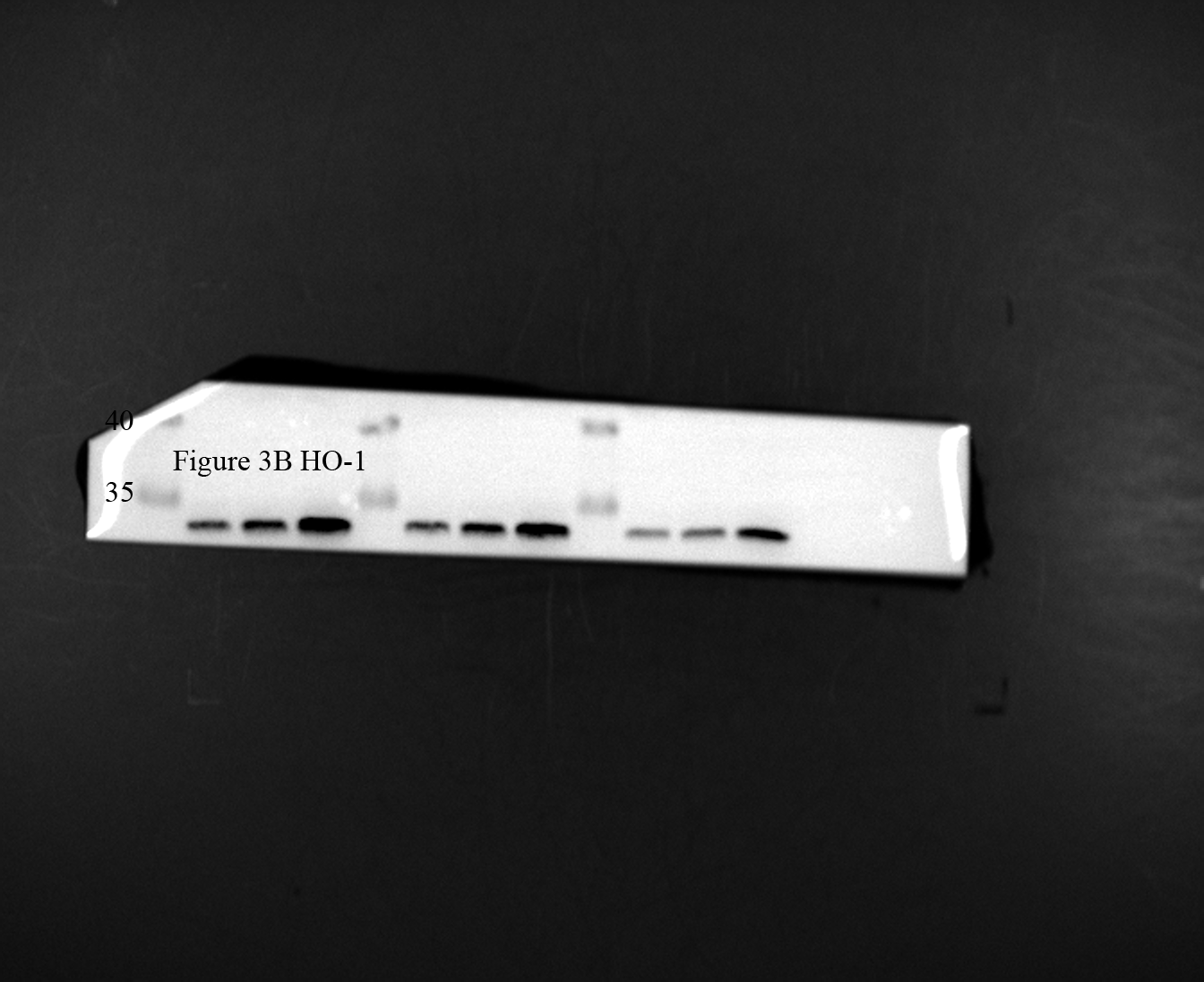

Supplement: Supplementary file 1 [file DataSheet1.zip › supplementary/Figure 3B HO-1.tif]

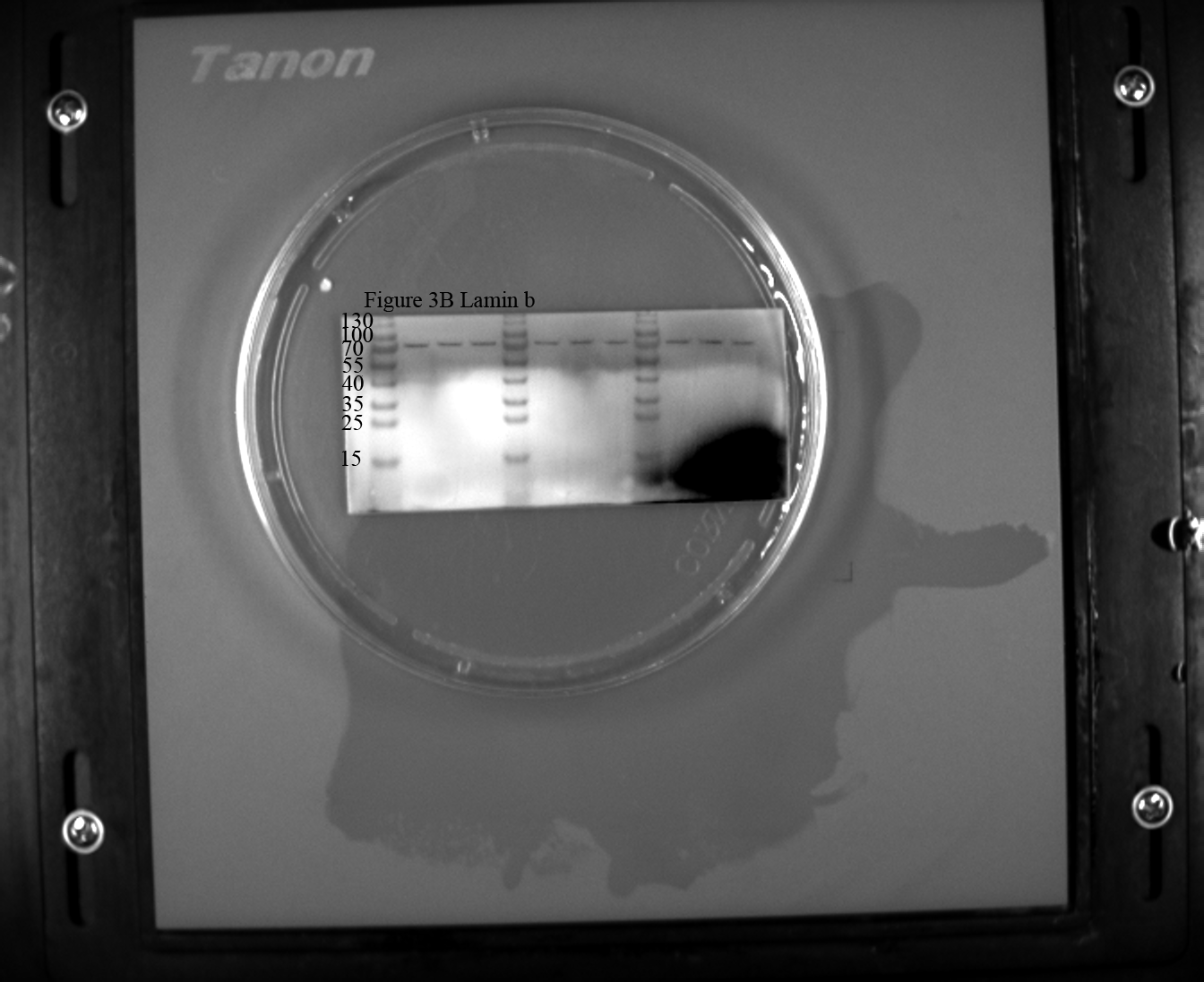

Supplement: Supplementary file 1 [file DataSheet1.zip › supplementary/Figure 3B Lamin b.tif]

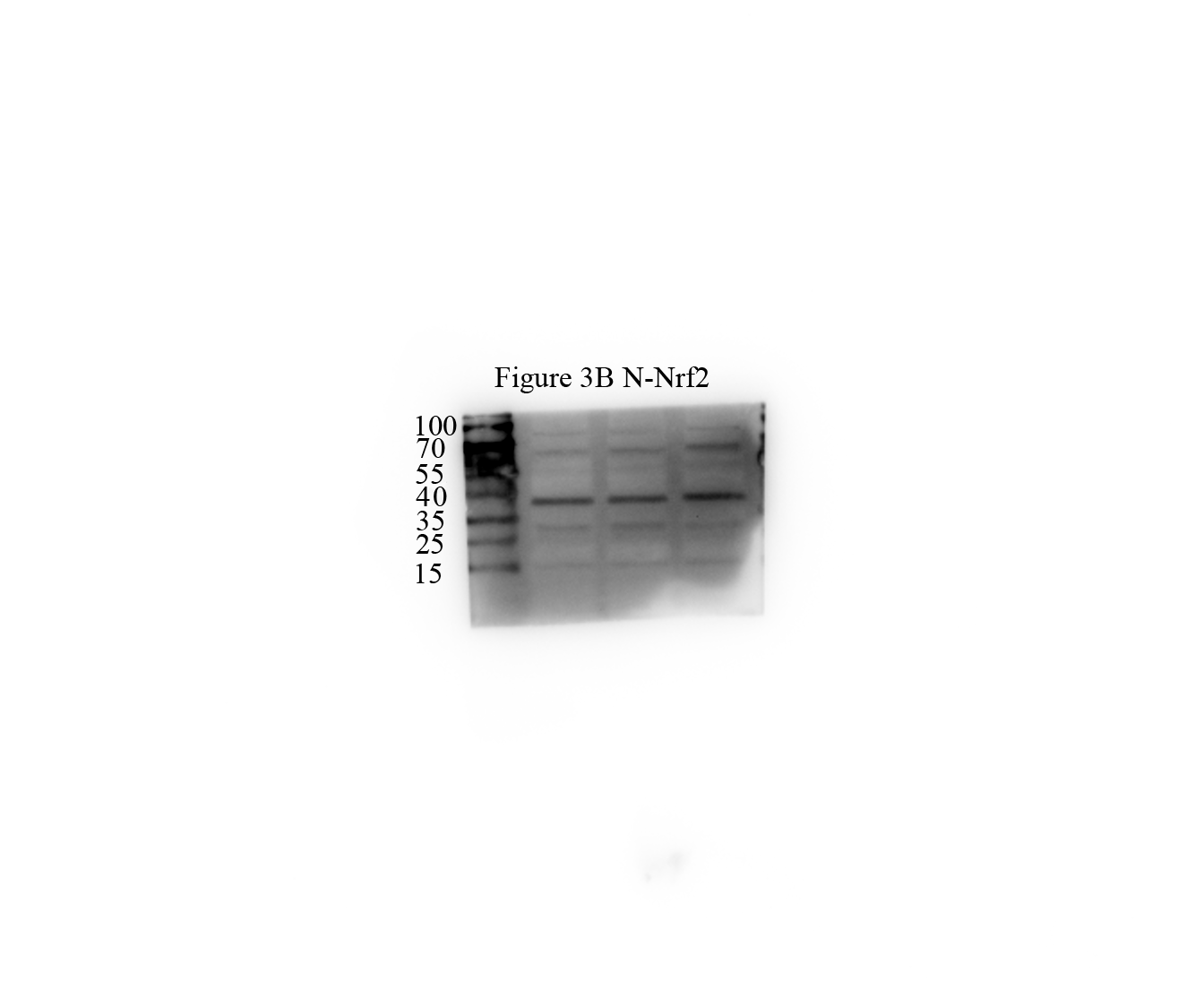

Supplement: Supplementary file 1 [file DataSheet1.zip › supplementary/Figure 3B N-Nrf2.tif]

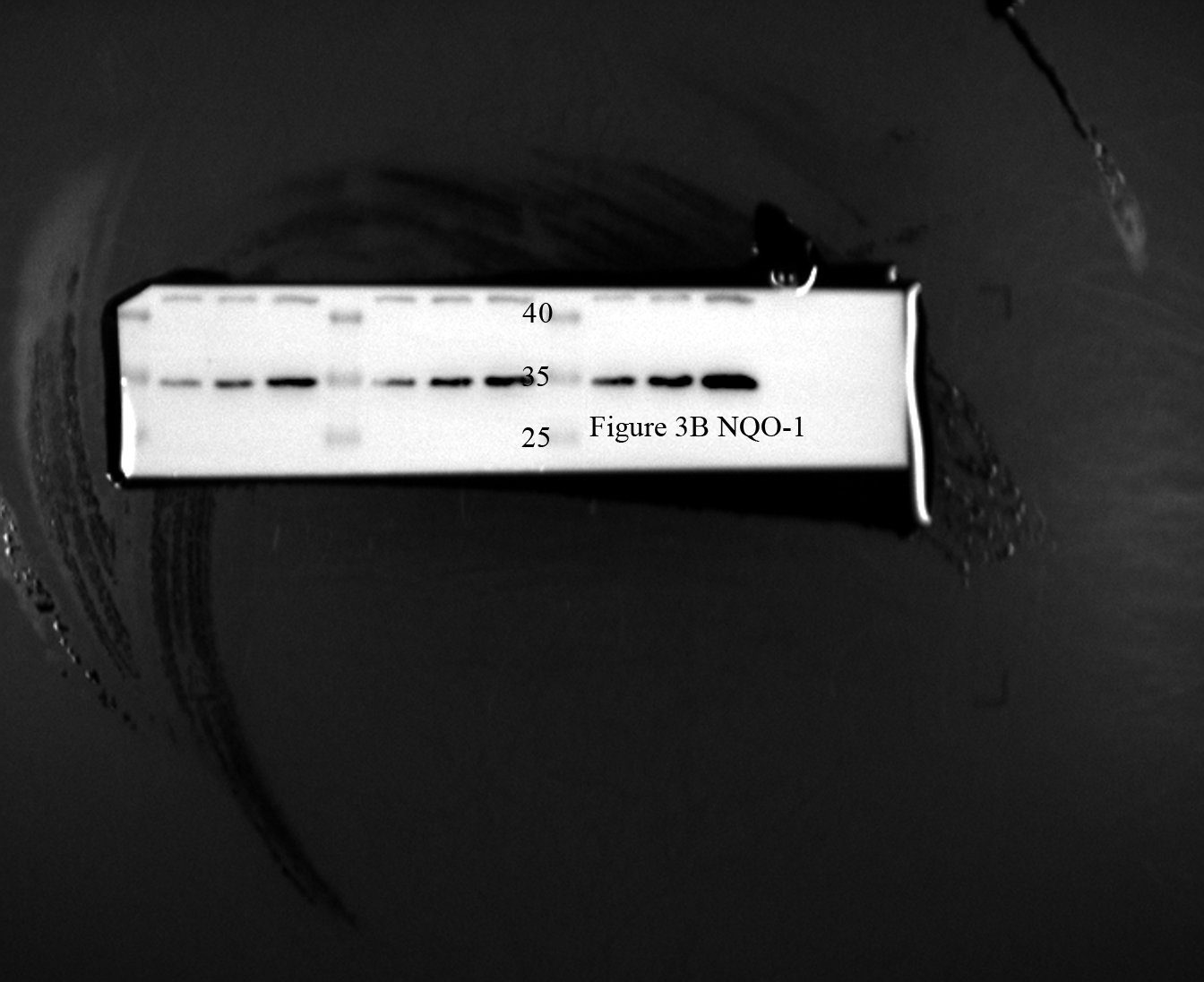

Supplement: Supplementary file 1 [file DataSheet1.zip › supplementary/Figure 3B NQO-1.tif]

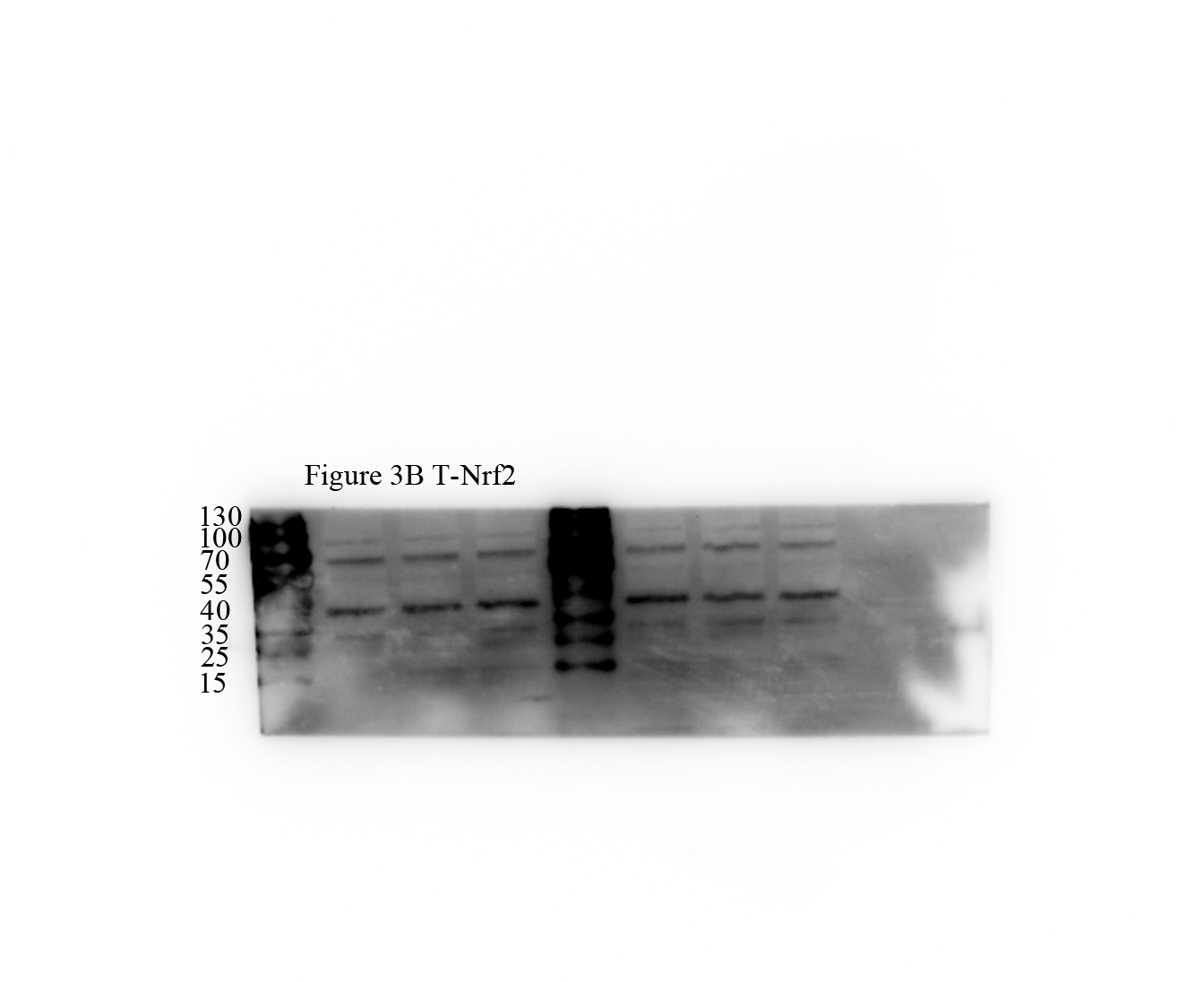

Supplement: Supplementary file 1 [file DataSheet1.zip › supplementary/Figure 3B T-Nrf2.tif]

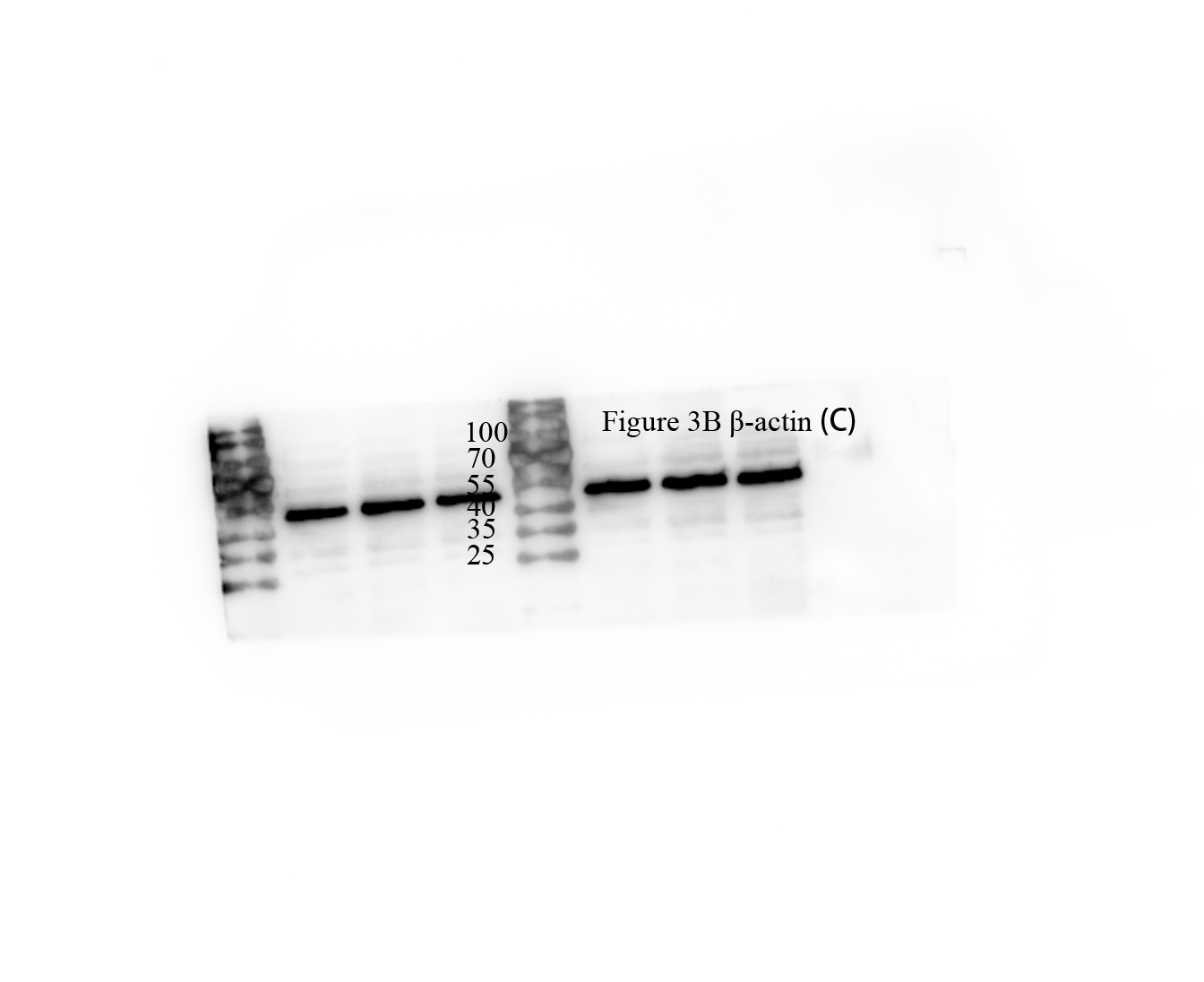

Supplement: Supplementary file 1 [file DataSheet1.zip › supplementary/Figure 3B β-actin (C).tif]

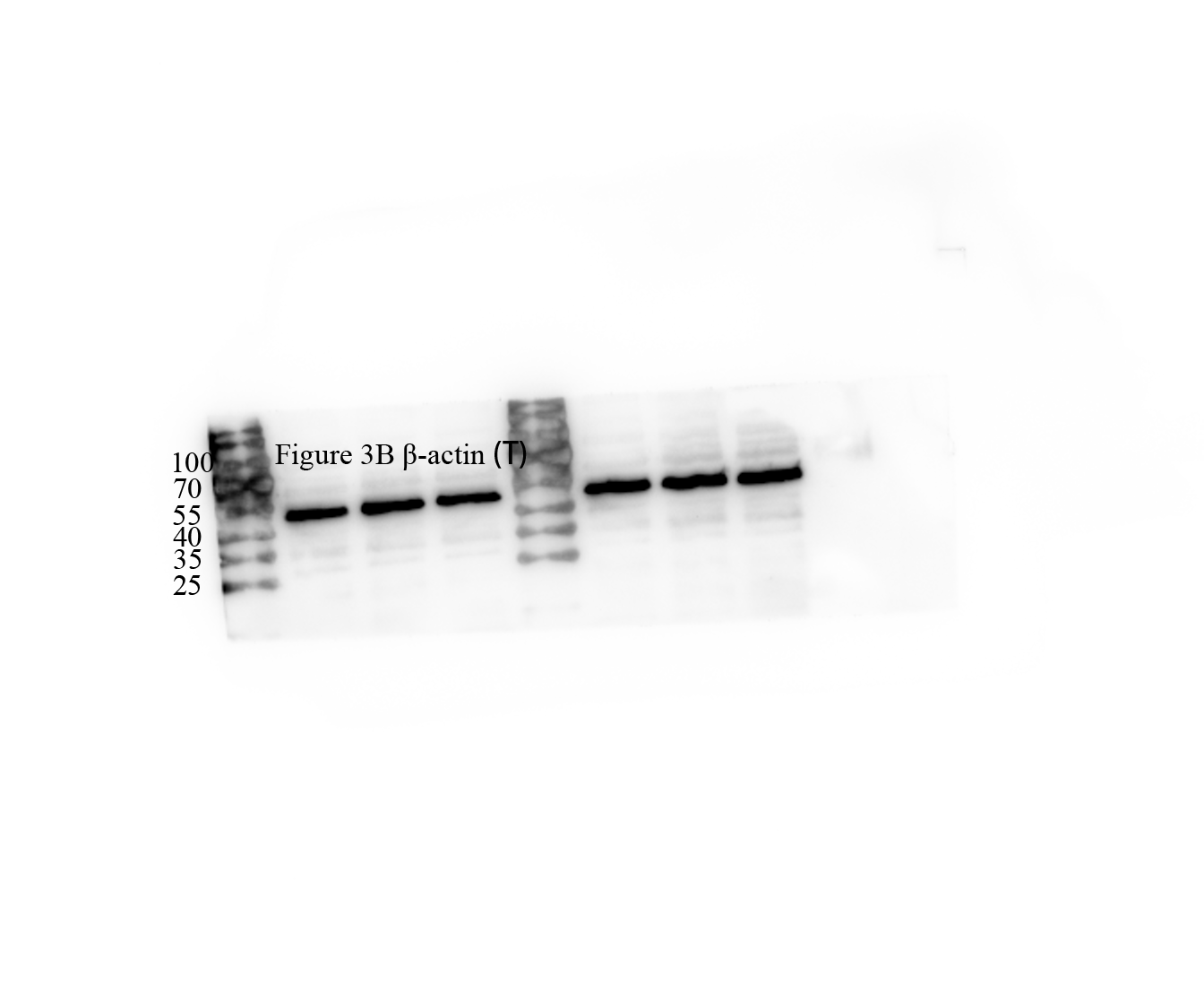

Supplement: Supplementary file 1 [file DataSheet1.zip › supplementary/Figure 3B β-actin (T).tif]

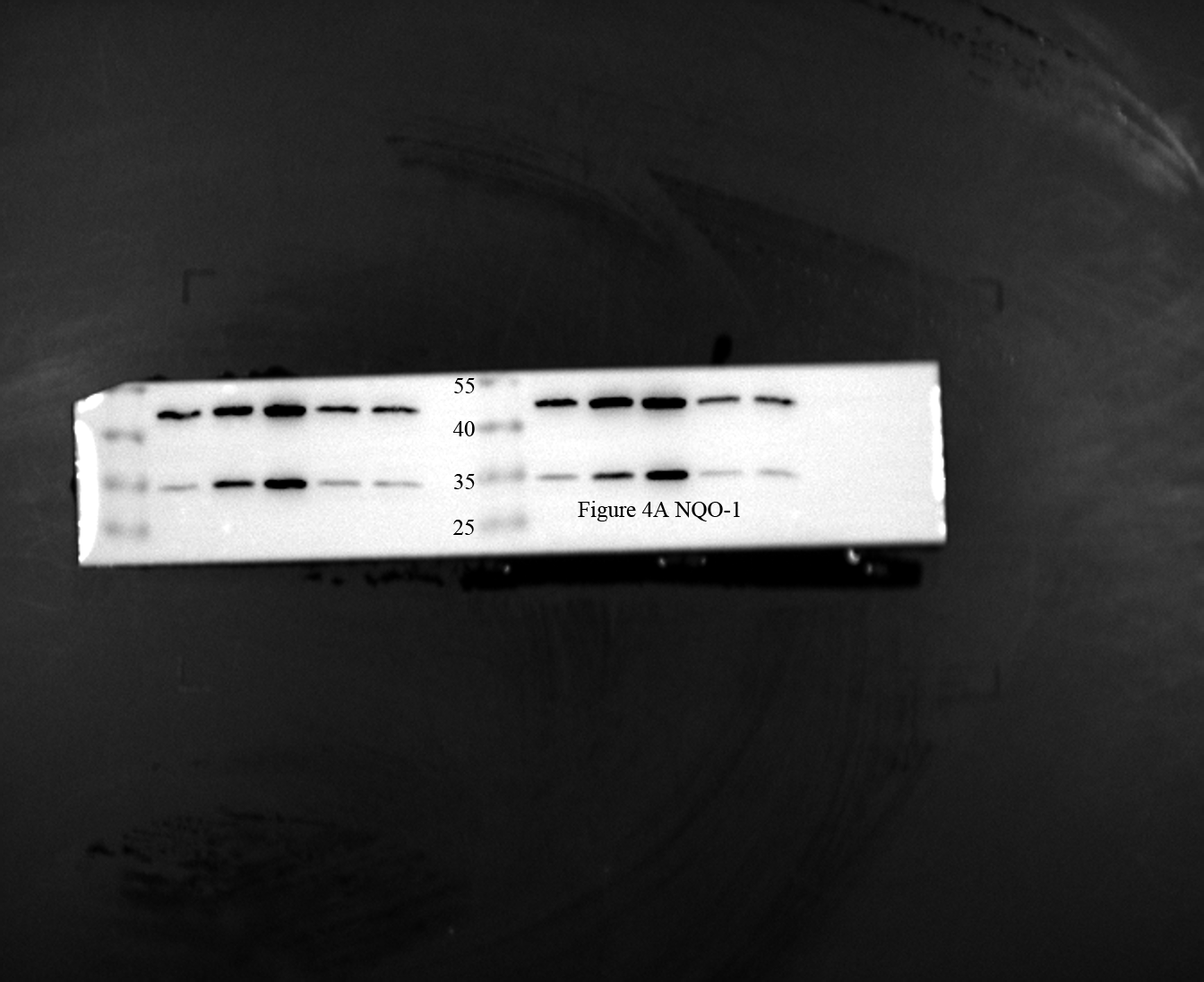

Supplement: Supplementary file 1 [file DataSheet1.zip › supplementary/Figure 4 A NQO-1.tif]

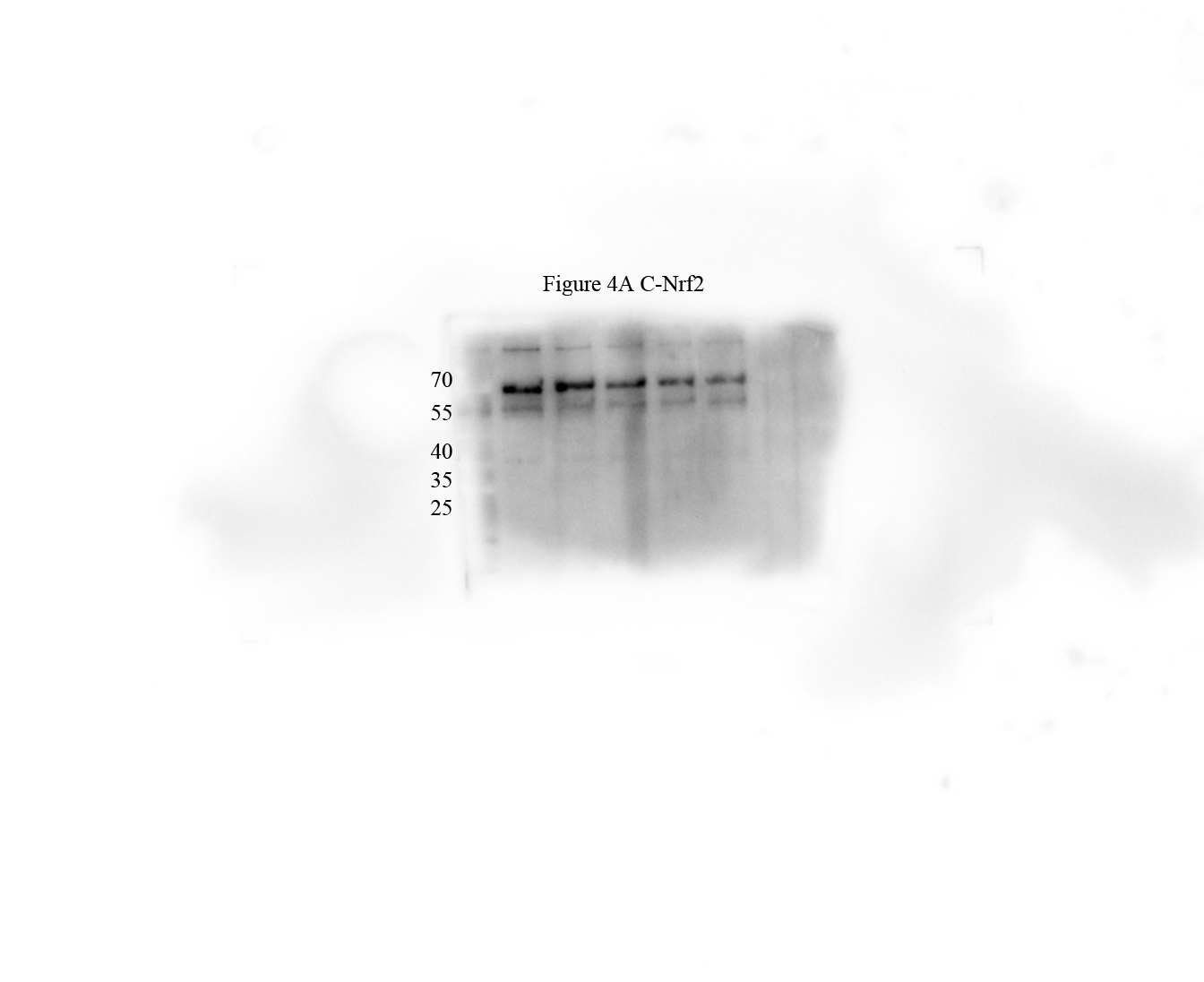

Supplement: Supplementary file 1 [file DataSheet1.zip › supplementary/Figure 4A C-Nrf2.tif]

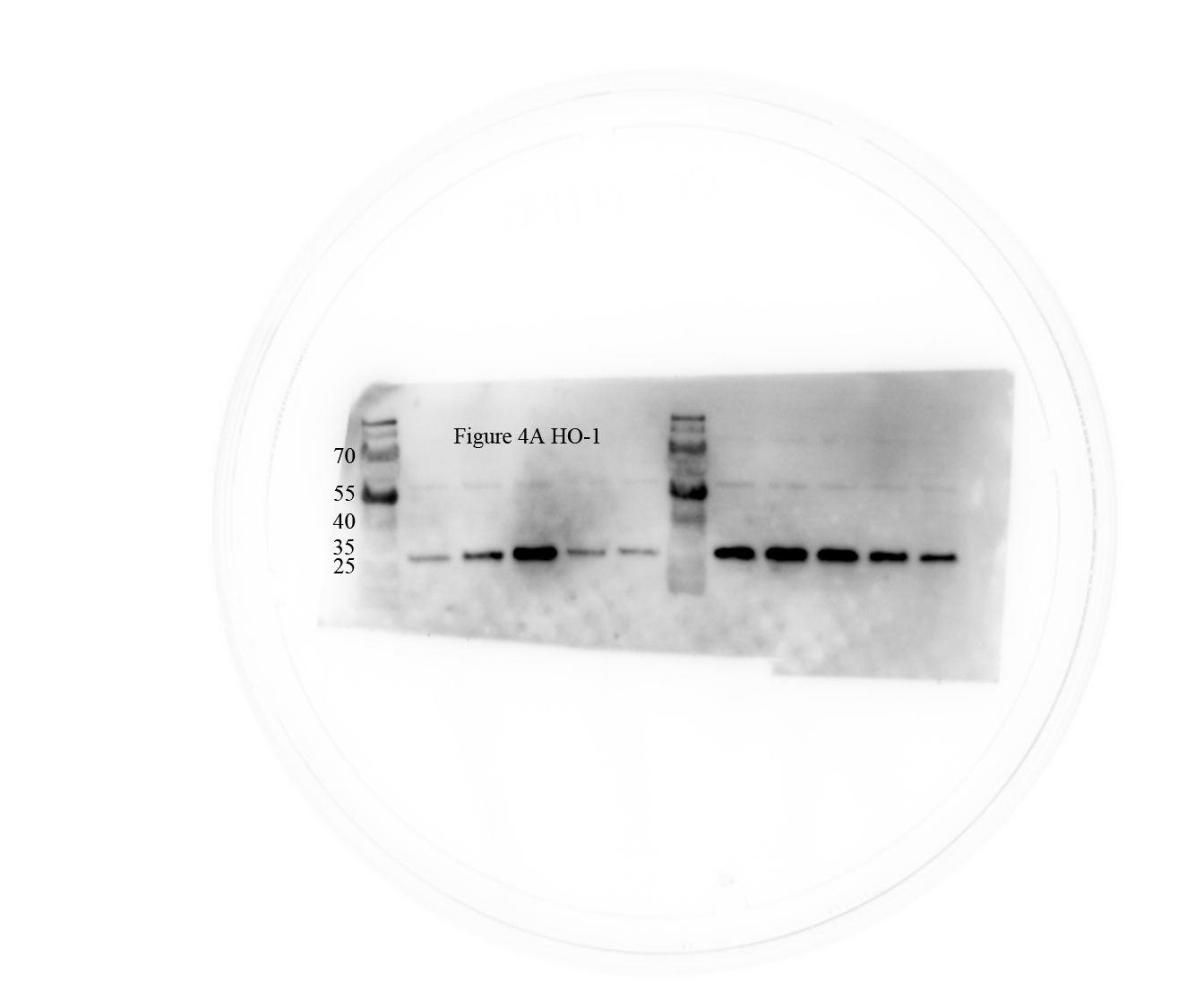

Supplement: Supplementary file 1 [file DataSheet1.zip › supplementary/Figure 4A HO-1.tif]

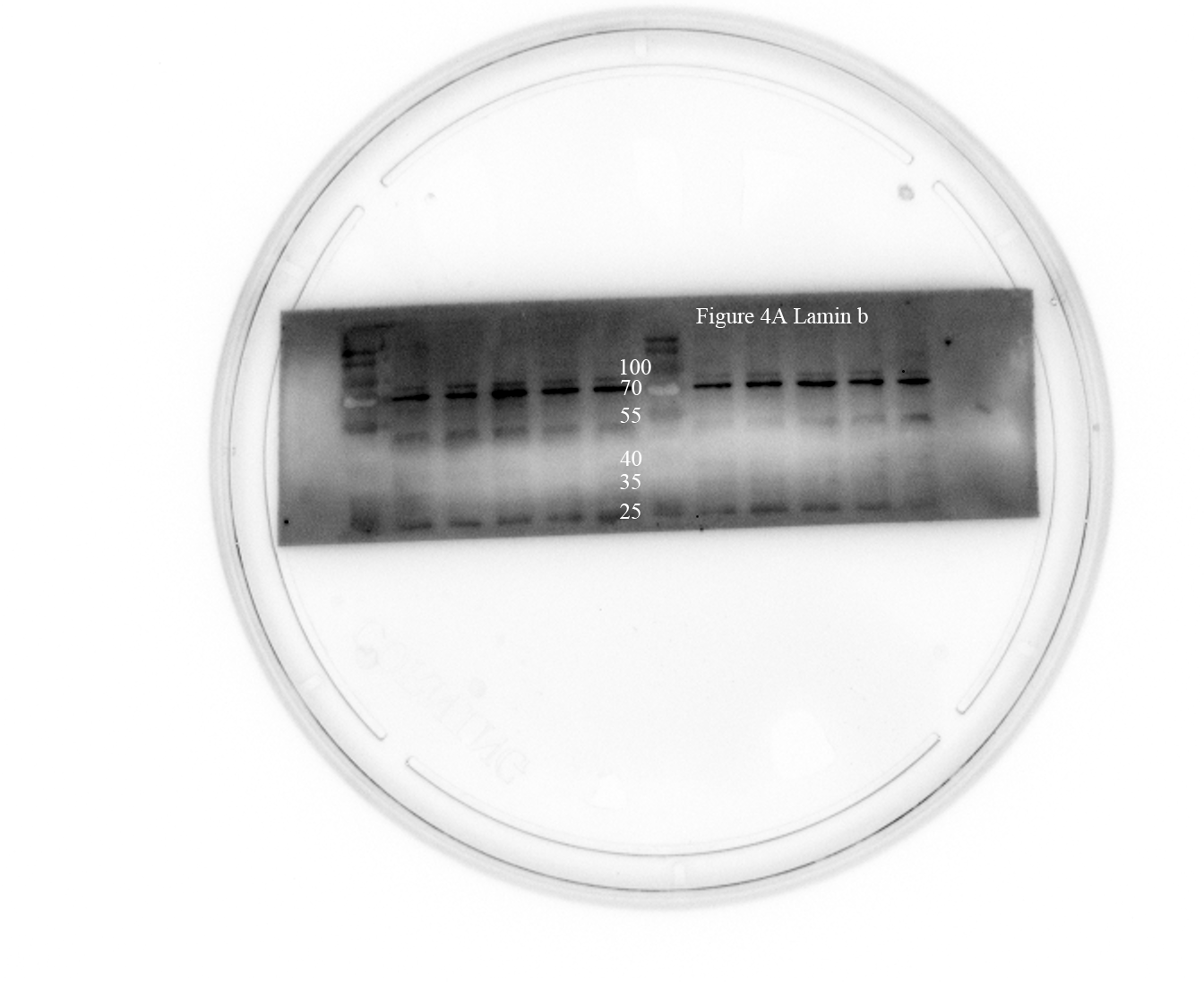

Supplement: Supplementary file 1 [file DataSheet1.zip › supplementary/Figure 4A Lamin b.tif]

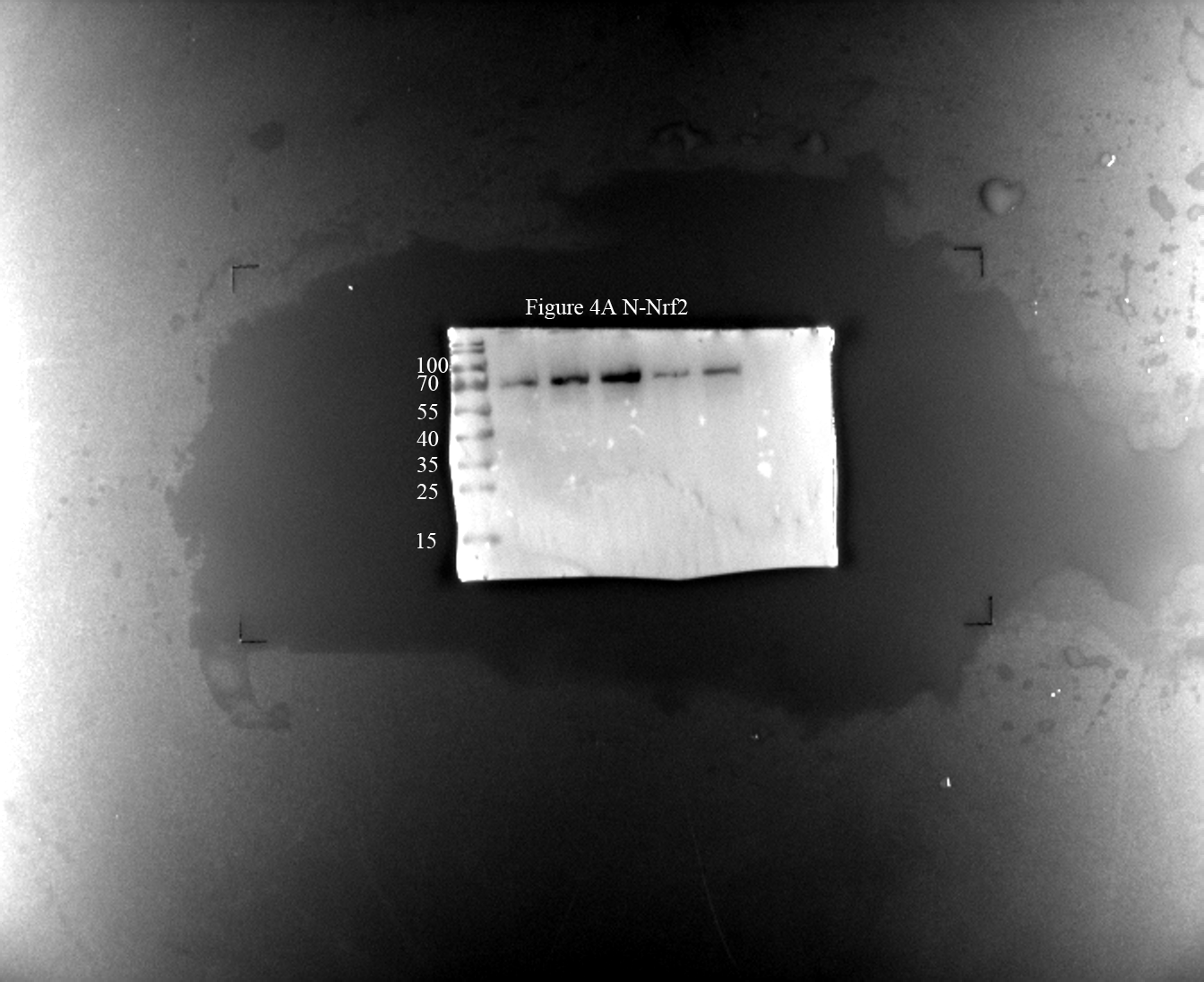

Supplement: Supplementary file 1 [file DataSheet1.zip › supplementary/Figure 4A N-Nrf2.tif]

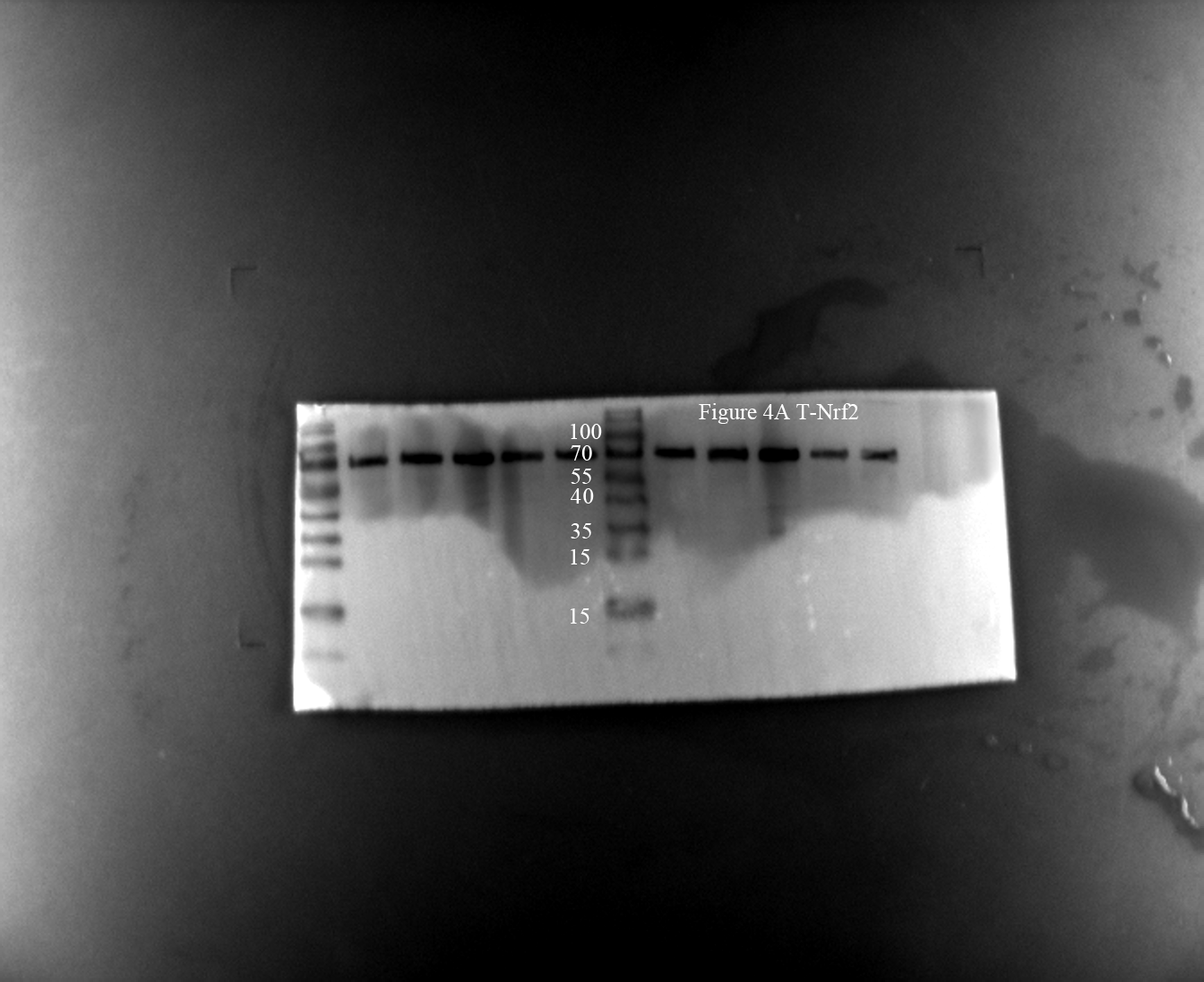

Supplement: Supplementary file 1 [file DataSheet1.zip › supplementary/Figure 4A T-Nrf2.tif]

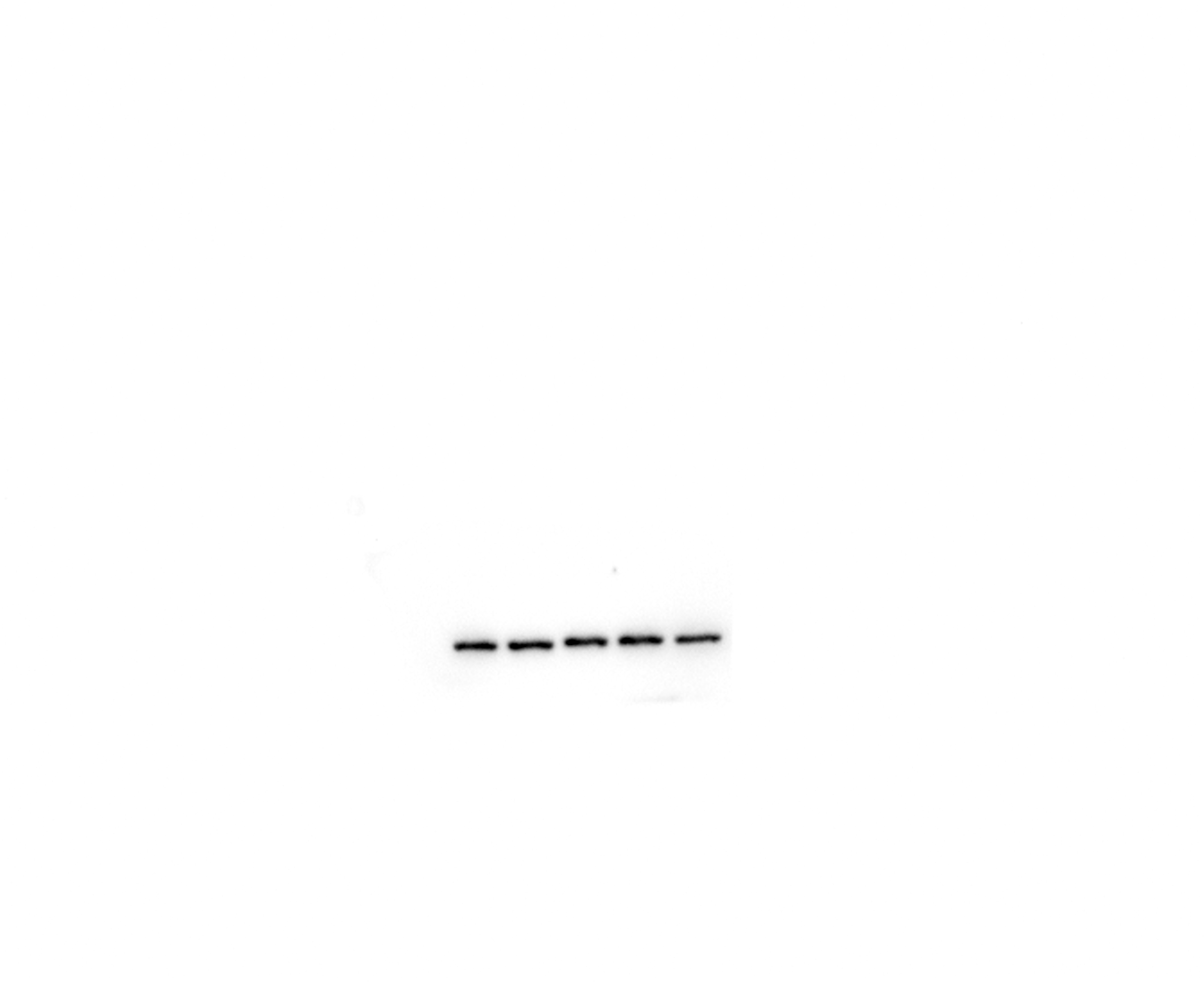

Supplement: Supplementary file 1 [file DataSheet1.zip › supplementary/Figure 4A β-actin (C).Tif]

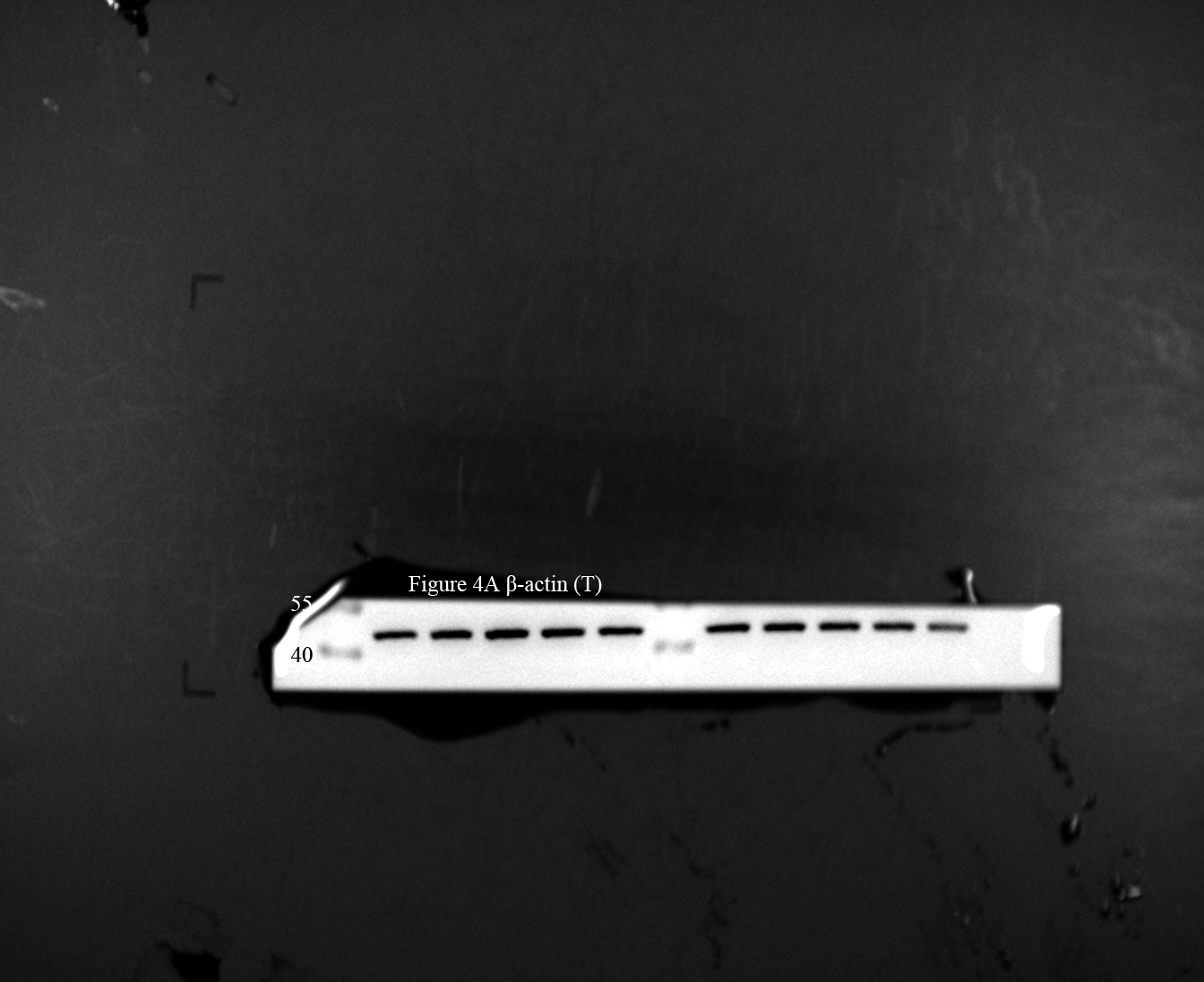

Supplement: Supplementary file 1 [file DataSheet1.zip › supplementary/Figure 4A β-actin (T).tif]

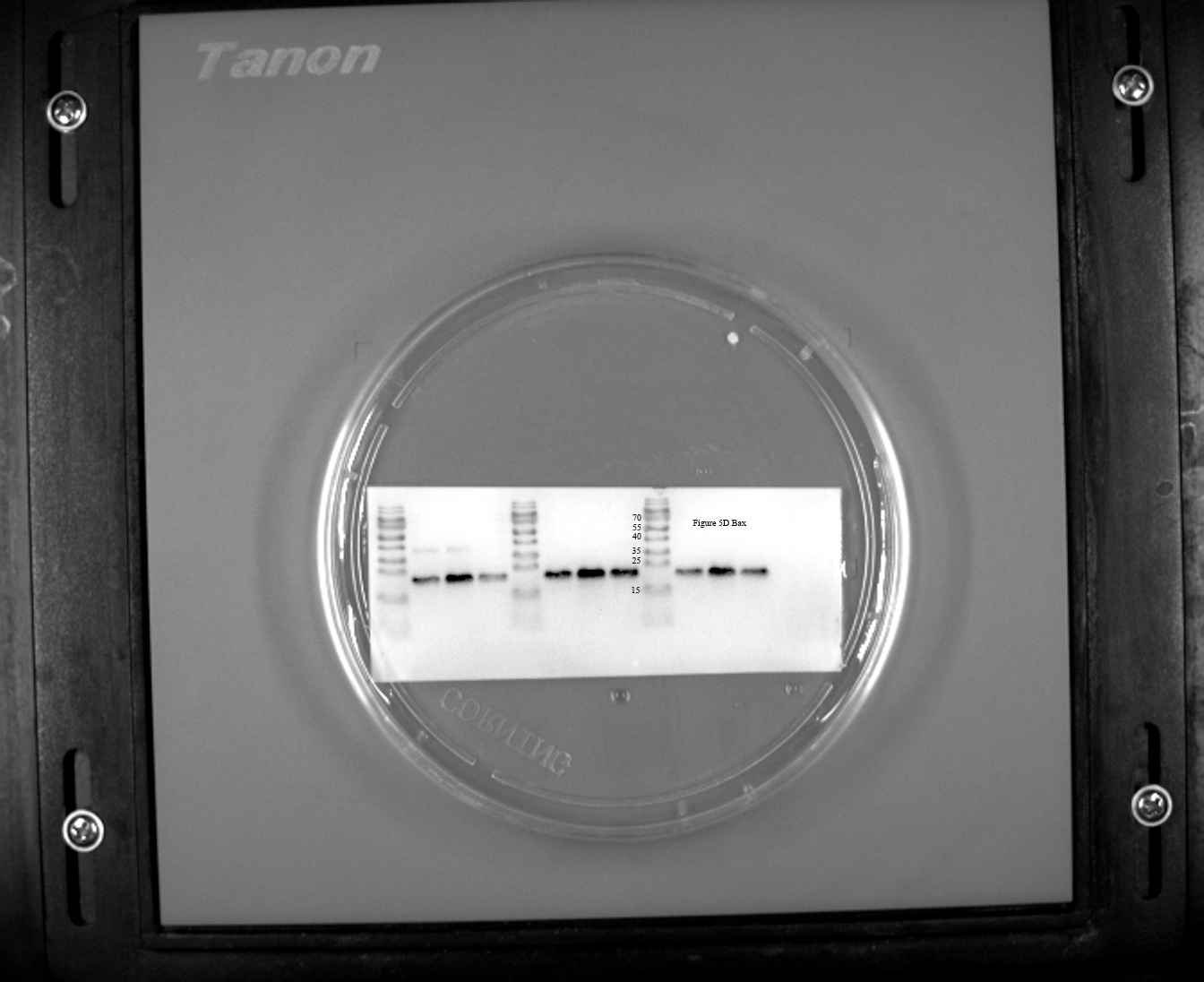

Supplement: Supplementary file 1 [file DataSheet1.zip › supplementary/Figure 5D Bax.tif]

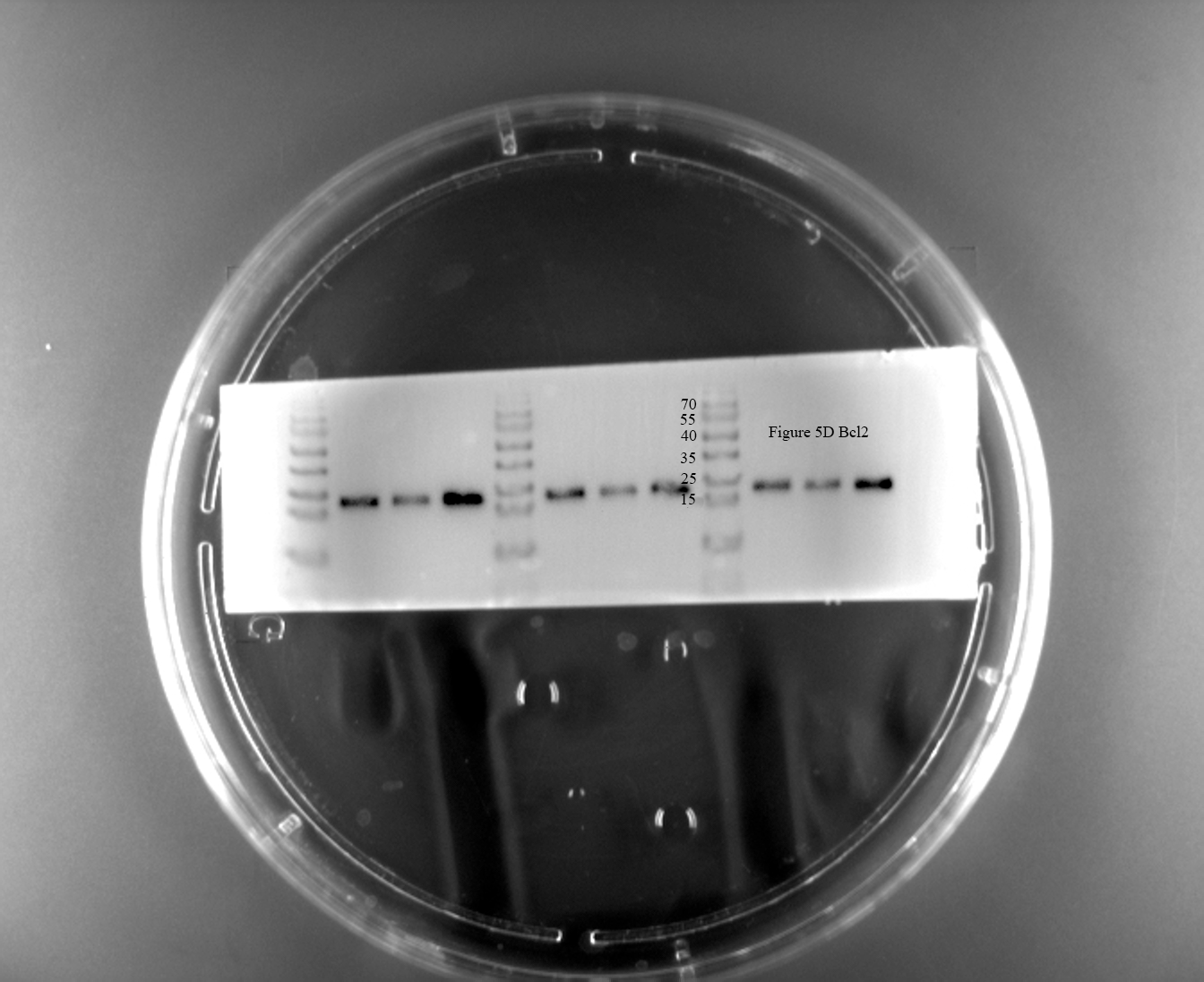

Supplement: Supplementary file 1 [file DataSheet1.zip › supplementary/Figure 5D Bcl2.tif]

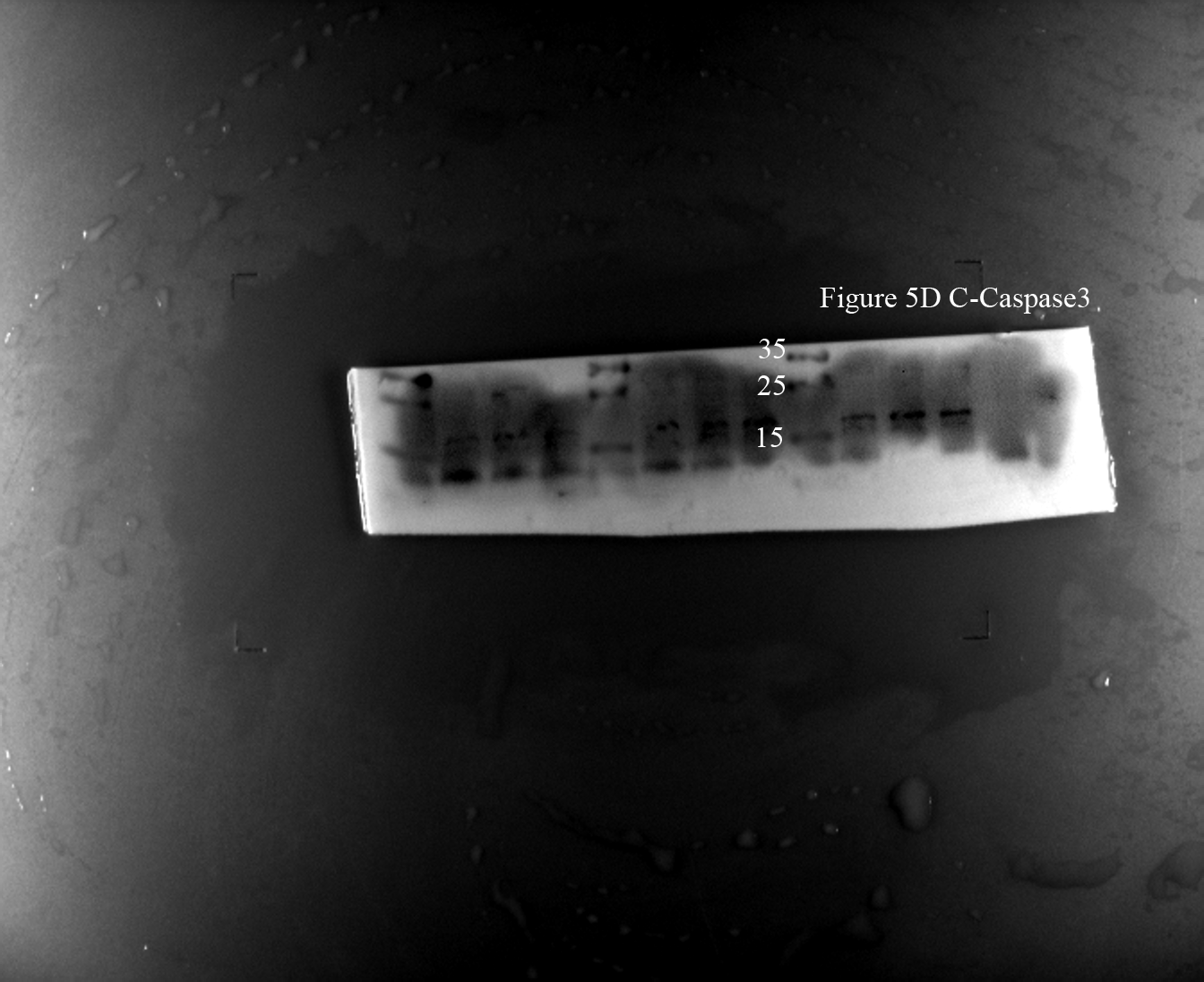

Supplement: Supplementary file 1 [file DataSheet1.zip › supplementary/Figure 5D c-caspase3.tif]

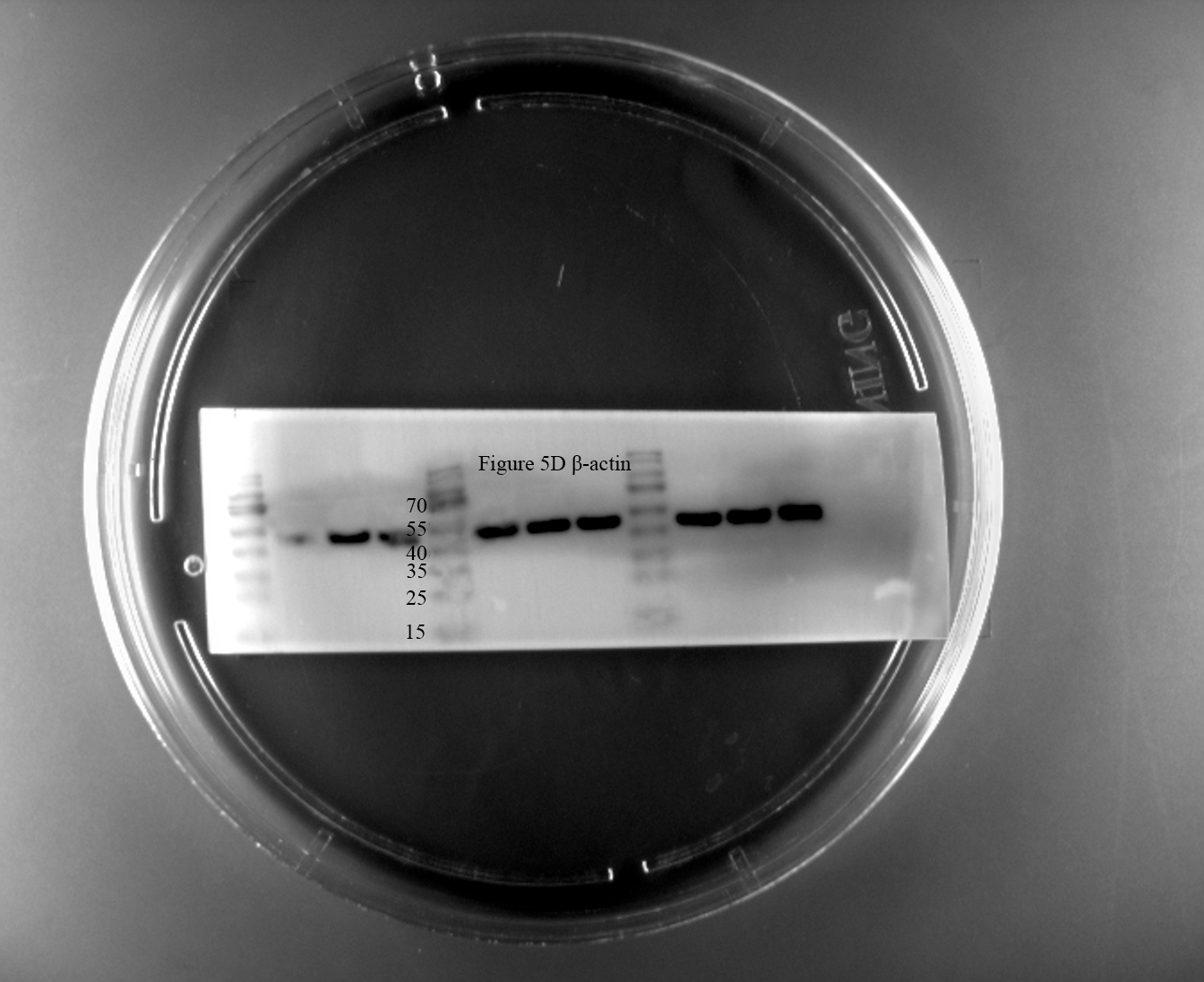

Supplement: Supplementary file 1 [file DataSheet1.zip › supplementary/Figure 5D β-actin.tif]

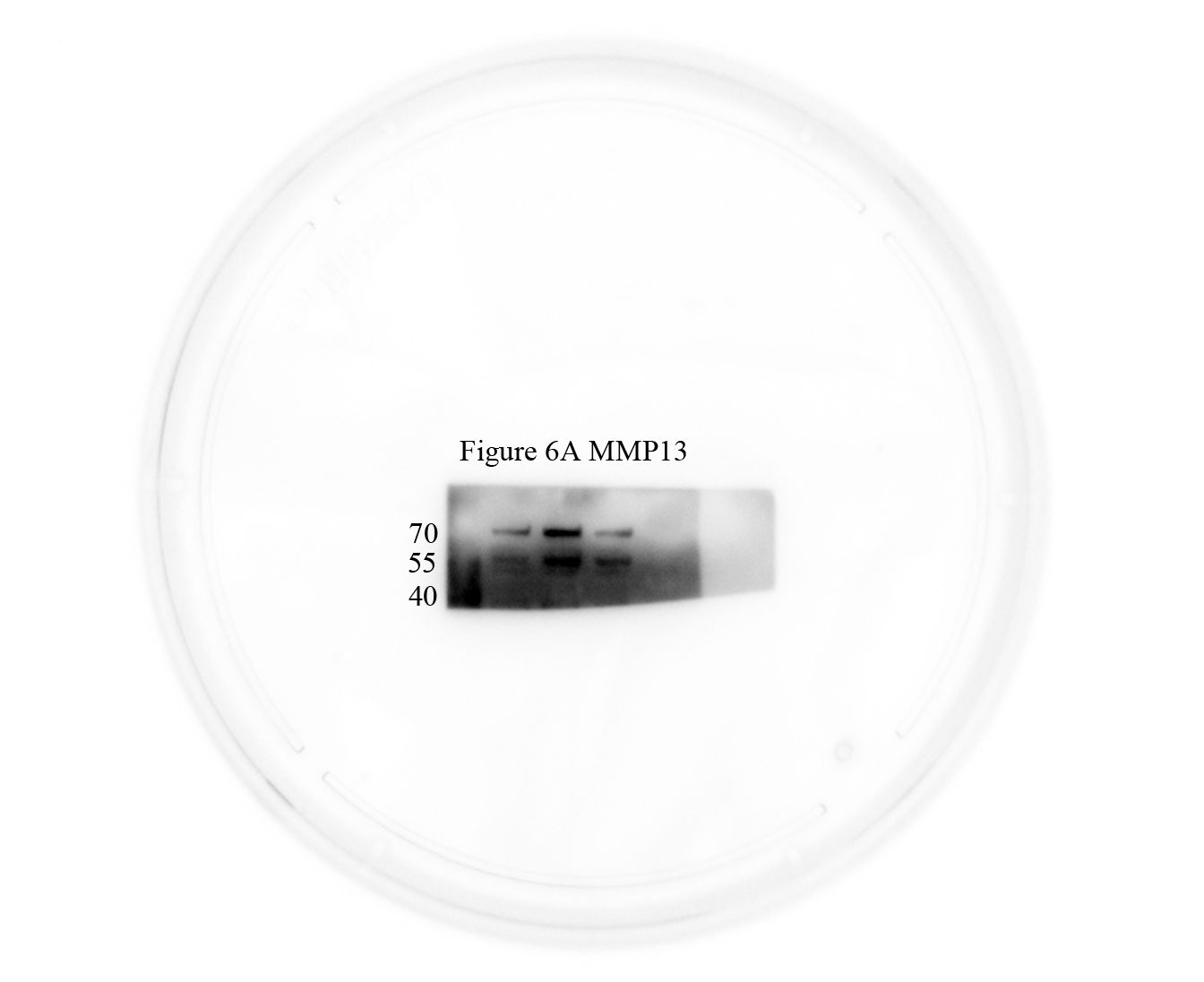

Supplement: Supplementary file 1 [file DataSheet1.zip › supplementary/Figure 6A MMP13.tif]

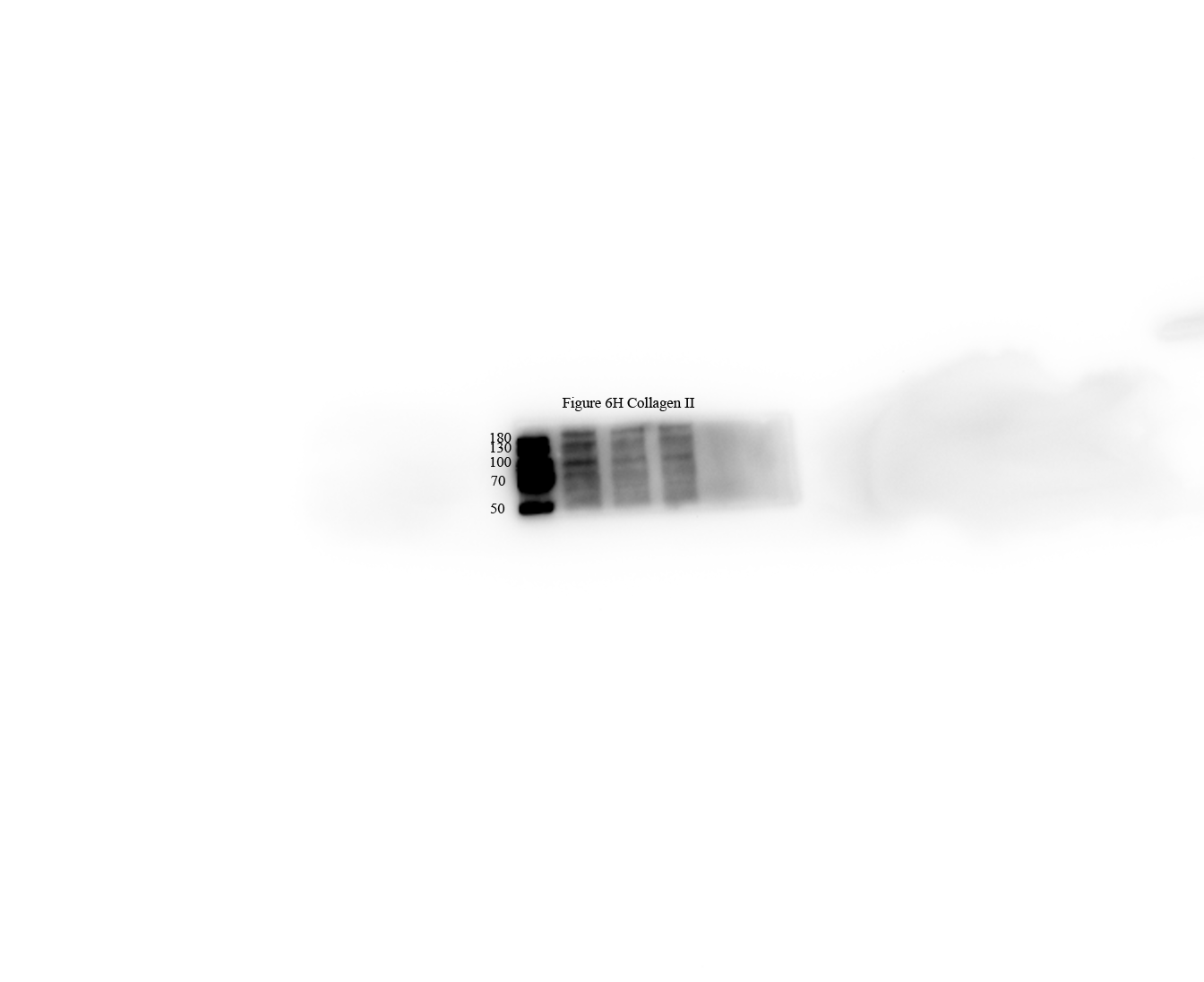

Supplement: Supplementary file 1 [file DataSheet1.zip › supplementary/Figure 6H Collagen II.tif]

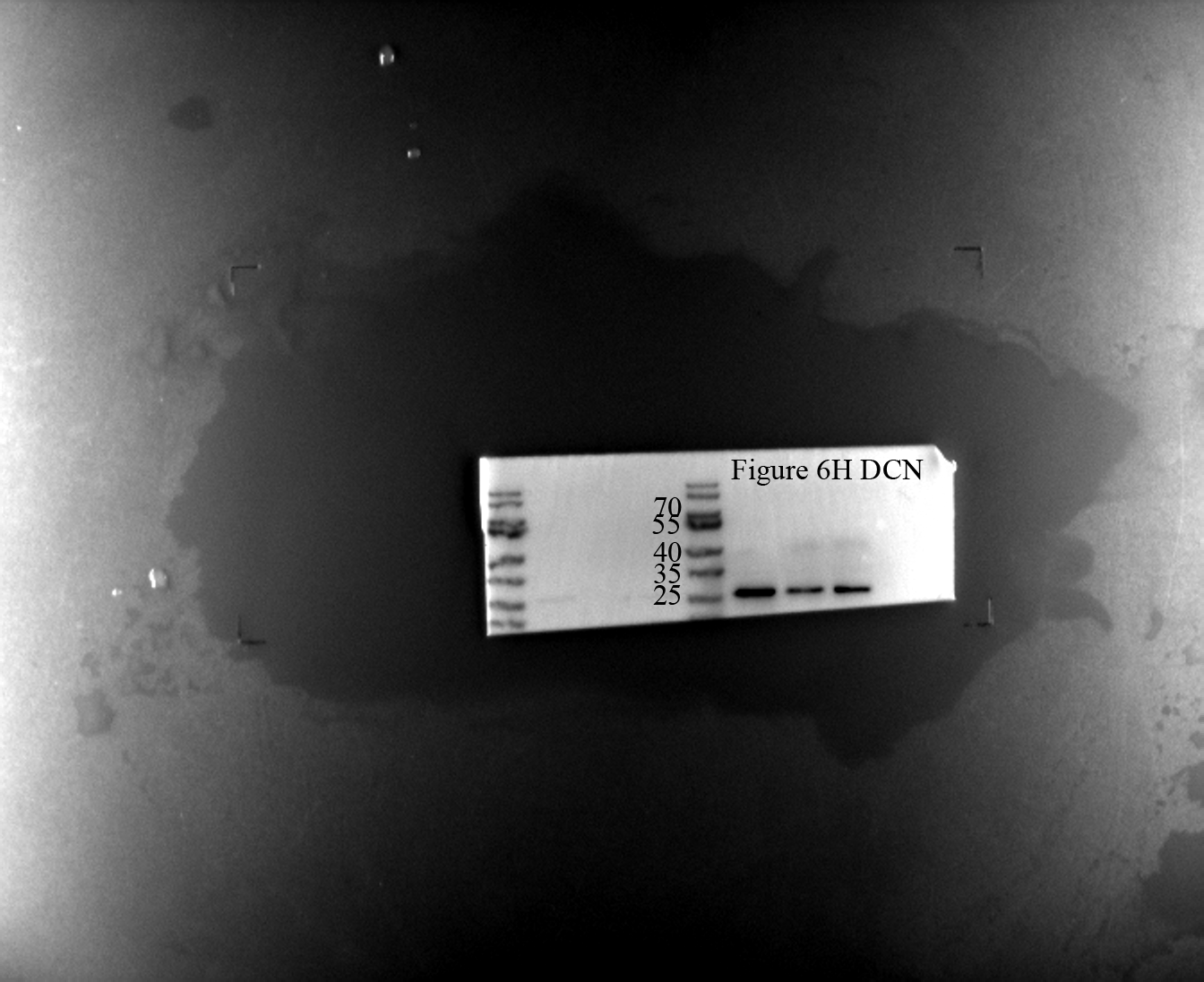

Supplement: Supplementary file 1 [file DataSheet1.zip › supplementary/Figure 6H DCN.tif]

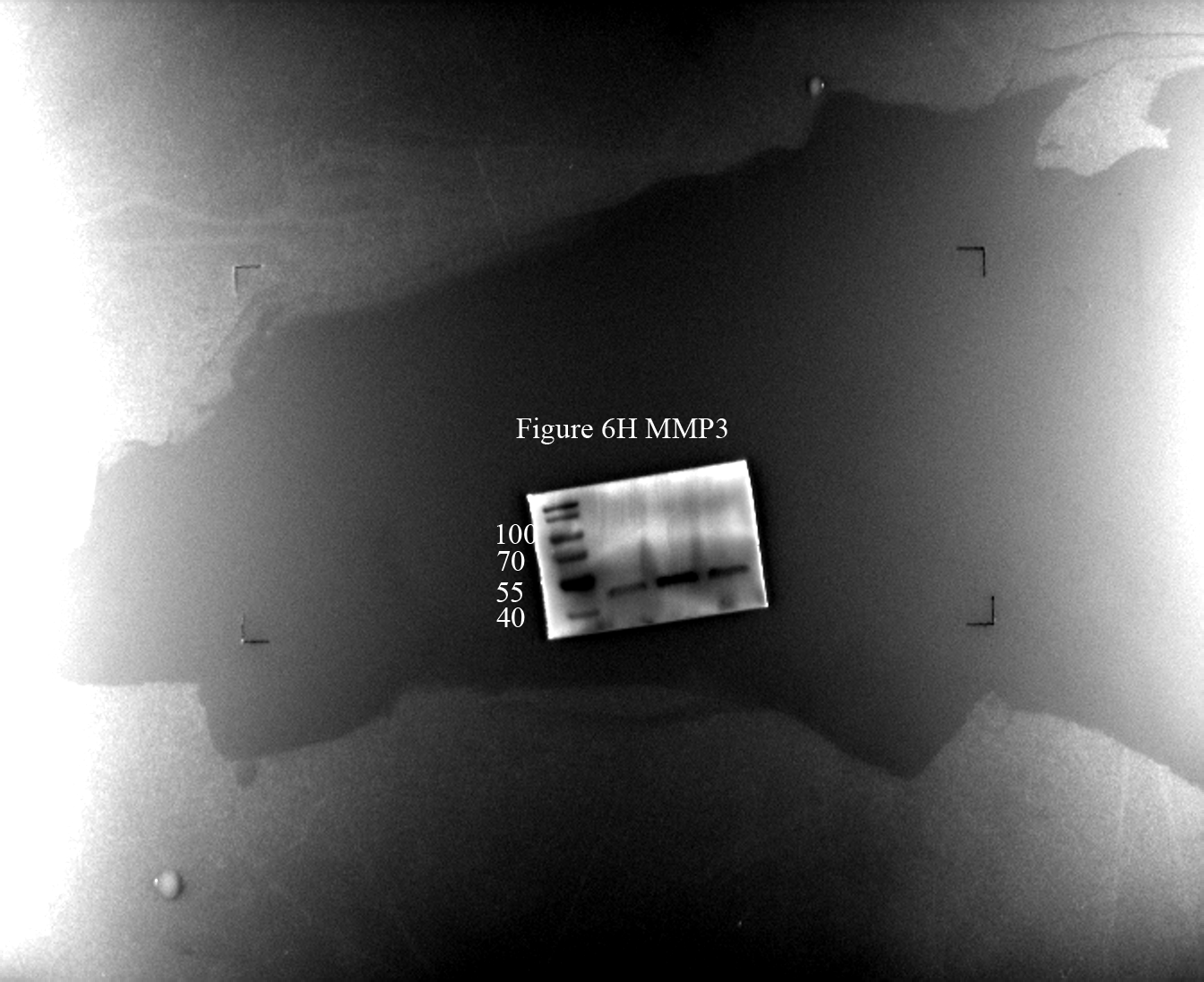

Supplement: Supplementary file 1 [file DataSheet1.zip › supplementary/Figure 6H MMP3.tif]

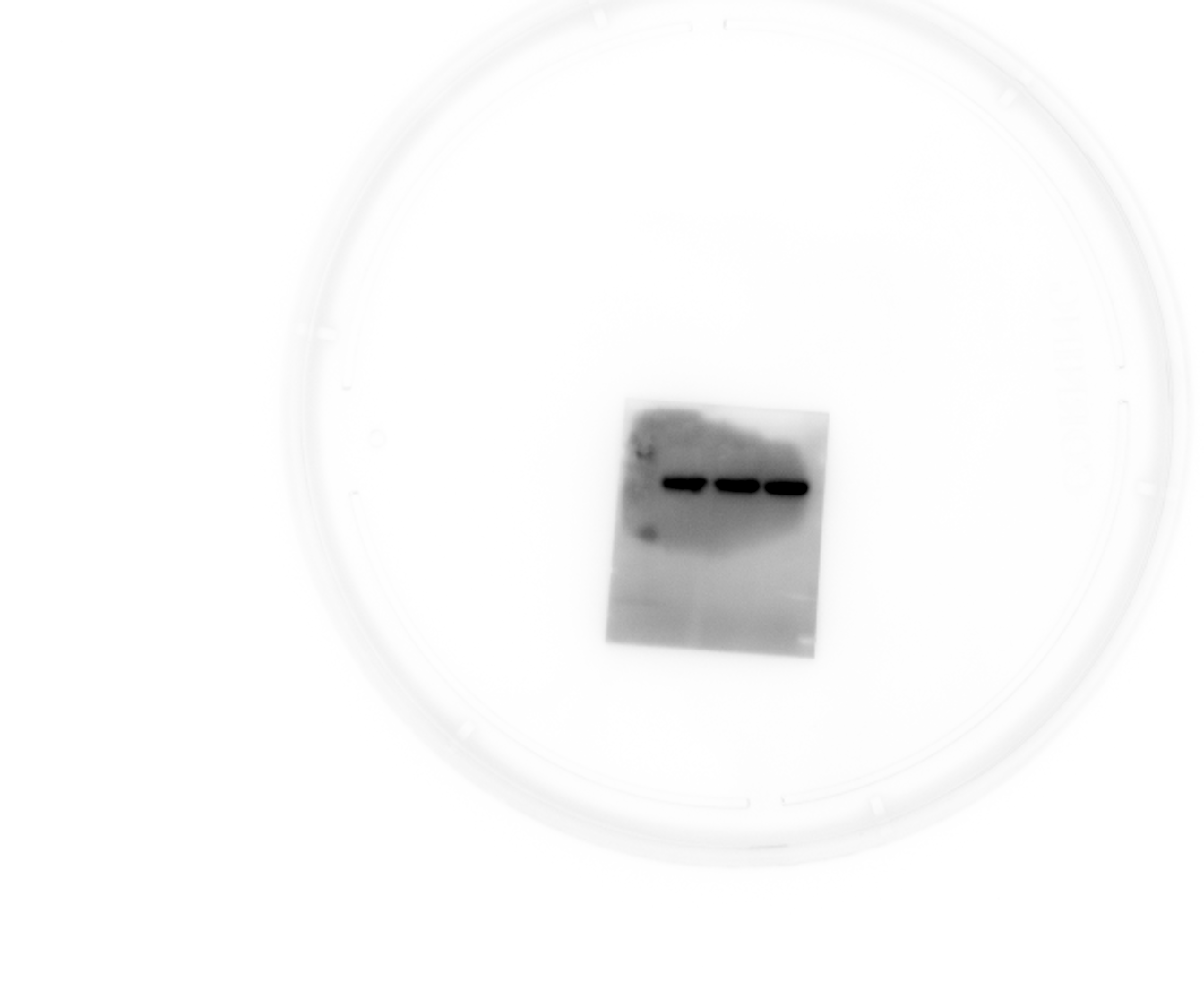

Supplement: Supplementary file 1 [file DataSheet1.zip › supplementary/Figure 6H β-actin.tif]

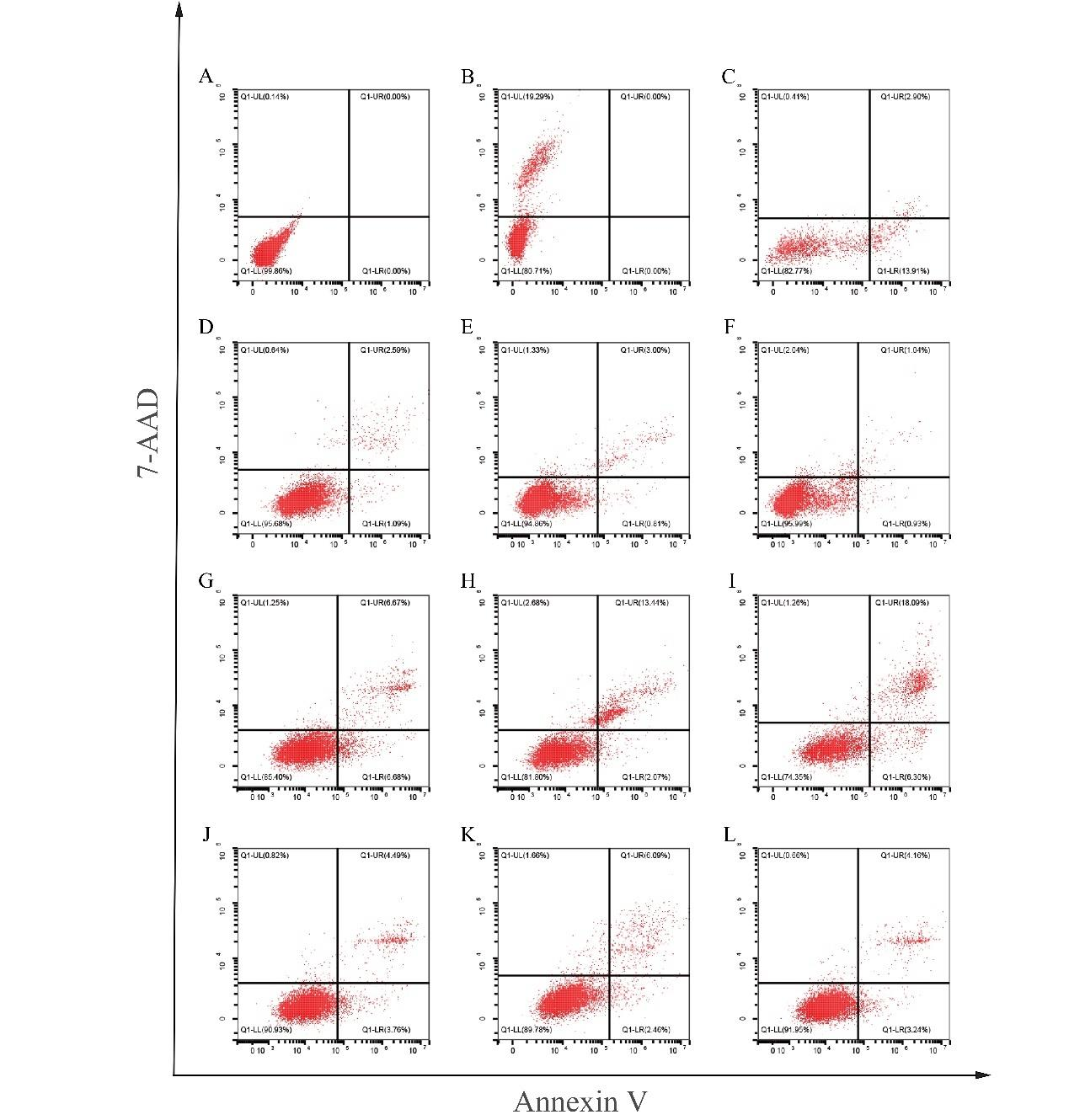

Supplement: Supplementary file 1 [file DataSheet1.zip › supplementary/figure s1.PNG]
